# Application of Ultraviolet-C Radiation and Gaseous Ozone for Microbial Inactivation on Different Materials

**DOI:** 10.1021/acsomega.2c05264

**Published:** 2022-11-15

**Authors:** Emmanuel I. Epelle, Andrew Macfarlane, Michael Cusack, Anthony Burns, William G. Mackay, Mostafa E. Rateb, Mohammed Yaseen

**Affiliations:** † School of Computing, Engineering & Physical Sciences, University of the West of Scotland, Paisley PA1 2BE, United Kingdom; ‡ School of Health & Life Sciences, University of the West of Scotland, Paisley PA1 2BE, United Kingdom; § ACS Clothing, 6 Dovecote Road Central Point Logistics Park, Glasgow ML1 4GP, United Kingdom

## Abstract

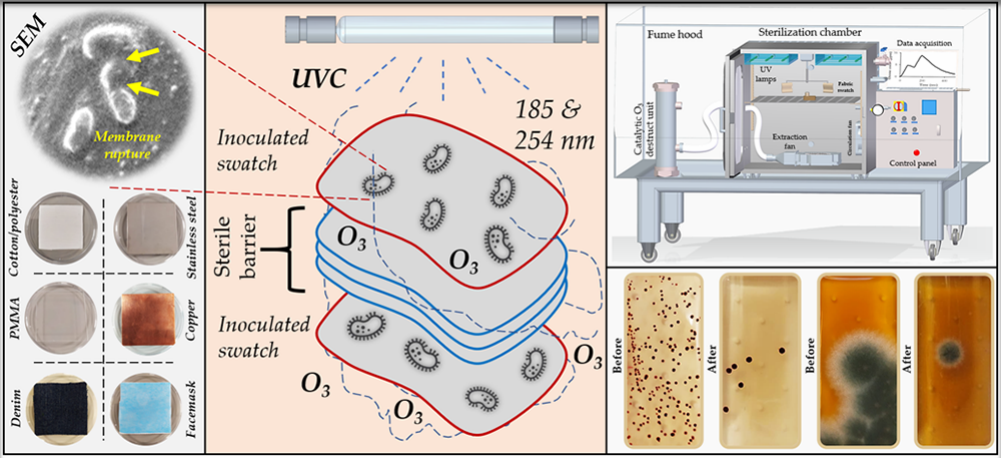

With the advent of the COVID-19 pandemic, there has been a global incentive for applying environmentally sustainable and rapid sterilization methods, such as ultraviolet-C radiation (UVC) and ozonation. Material sterilization is a requirement for a variety of industries, including food, water treatment, clothing, healthcare, medical equipment, and pharmaceuticals. It becomes inevitable when devices and items like protective equipment are to be reused on/by different persons. This study presents novel findings on the performance of these sterilization methods using four microorganisms (*Escherichia coli,Staphylococcus aureus,Candida albicans,* and *Aspergillus fumigatus*) and six material substrates (stainless steel, polymethyl methacrylate, copper, surgical facemask, denim, and a cotton-polyester fabric). The combination of both ozone and UVC generally yields improved performance compared to their respective applications for the range of materials and microorganisms considered. Furthermore, the effectiveness of both UVC and ozone was higher when the fungi utilized were smeared onto the nonabsorbent materials than when 10 μL droplets were placed on the material surfaces. This dependence on the contaminating liquid surface area was not exhibited by the bacteria. This study highlights the necessity of adequate UVC and ozone dosage control as well as their synergistic and multifunctional attributes when sterilizing different materials contaminated with a wide range of microorganisms.

## 1 Introduction

During the outbreak of infectious diseases such as the coronavirus (COVID-19), personal protective equipment (PPE) is key for the safety of healthcare workers and the general population against a diverse range of contamination sources.^1,2^ At the early stages of the COVID-19 pandemic, the unprecedented demand for PPE (including surgical masks, coveralls, respirators, and goggles), by healthcare facilities and the public, was the main incentive for further developments and the increased application of decontamination procedures, to enable their reuse.^3−5^ However, the environmental impact, as a consequence of the increased amounts of generated solid waste from these materials, cannot be overlooked and requires adequate management.^6^ The textile industry is the second largest polluter of the environment contributing up to 8–10% of the global CO_2_ emissions and approximately 20% of the global waste.^7,8^ It is also worth pointing out that fiber manufacturing processes, which utilize synthetic fibers (such as polyester and polypropylene, which are predominantly applied for PPE manufacturing), require high amounts of energy. The review by Karim et al.^8^ pointed out that doubling a garment’s (non-PPE clothing) lifetime can reduce greenhouse gas (CHG) emissions by 44%, with an annual economic saving of up to $460 billion. More specifically, the reuse of medical apparel has the potential of reducing GHG emissions, energy consumption, water consumption, and solid waste generation by 66, 64, 83, and 84%, respectively.^9^

Besides PPE decontamination, reusable medical devices often made from a variety of polymers (e.g., polycarbonate, polyurethane, and polymethyl methacrylate (PMMA)) are primary candidates for decontamination in clinical settings. The removal of biofilm-forming bacteria from hard surfaces (e.g., stainless steel) in the food industry is also paramount to food processing safety. The review by Varga and Szigeti^10^ presents an extensive discussion of decontamination methods applicable to the dairy industry with a focus on ozone, whereas a general account of sterilization techniques is presented in the work of Rogers.^11^ Several experimental studies have demonstrated the effects of a variety of sterilization methods, including plasma, gamma irradiation, ultraviolet irradiation (of type C), dry and moist heat, steam, hydrogen peroxide (gas and liquid), microwave, ozone (gas and liquid), peracetic acid, ethanol, glutaraldehyde, orthophthalaldehyde (OPA), ethylene oxide, benzalkonium chloride, and hypochlorite.^5,12,13^ The performance of these methods for diverse applications has mainly been assessed using factors such as decontamination efficacy, cycle time, penetration capability, substrate/material compatibility, operational safety, cost of implementation, and environmental sustainability, with an overwhelming majority focusing on the decontamination efficacy.

Heat-based sterilization at 134°C has been shown to damage a filter material’s microstructure^14^ while also damaging other heat-sensitive regions of protective equipment. A high concentration of liquid hydrogen peroxide during plasma sterilization may neutralize the electrostatic charge of the equipment (a key feature required for moisture resistance properties).^3,15^ Ethylene oxide, a key disinfectant in hospitals, is less environmentally friendly and carcinogenic;^11^ similar challenges are also posed by chlorine-based disinfectants.^16^ Compared to ozone, hydrogen peroxide vapor has a lower penetration efficiency,^11^ with a lower oxidative potential^17^ for rapid disinfection. This high reactivity of ozone may be disadvantageous for some applications where certain ozone-degrading polymers are required/utilized. However, polymers such as polystyrene are capable of absorbing dry gaseous ozone and releasing it efficiently for biocidal action.^18^ UVC treatment may be affected by poor penetration, particularly when sterilizing materials of complex geometries, and may require extra/time-consuming preparatory steps^3,19,20^ for enhanced penetration prior to the main decontamination cycle; they must also be reflected in many directions for increased effectiveness on the substrate.^2,21^ However, their versatile functionality, besides decontamination, makes them attractive for several other applications.^22−28^Table 4 summarizes key merits and demerits of both decontamination methods, particularly during their large-scale application.

A recent review by Rubio-Romero et al.^4^ concluded that the most promising methods for the disinfection and sterilization of PPE include those that use hydrogen peroxide vapor, ozone gas, and UVC radiation; other methods were not fully recommended. This review also demonstrated a significantly higher number of published studies on the application of hydrogen peroxide vapor relative to ozone. This may be attributed to the fact that a majority of ozone sterilization studies in the literature have focused on water treatment^29^ compared to textiles, polymers, and other materials; this observation has motivated the work herein. Recently, gaseous ozone (20 ppm for 40 min) has been successfully (4 log reduction) applied for the treatment of facemasks contaminated with the influenza-A virus, but this has been accompanied with damage to the elastic bands.^30^ The efficacy of gaseous ozone disinfection (56 ppm, 40–240 min) has also been demonstrated in the work of Ljungberg,^31^ where several medical devices contaminated with *Geobacillus stearothermophilus* spores were treated. A similar study by Thill and Spaltenstein^32^ analyzed the gaseous ozone (100–1000 ppm) disinfection efficiency of medical devices using a bespoke chamber and achieved a 12 log reduction of *G. stearothermophilus.* Hudson et al.^33^ using gaseous ozone showed that 20–25 ppm of ozone (at RH > 90%) was able to inactivate (>3 log reduction) the Murine Coronavirus (MCV) on different adsorbent and nonadsorbent surfaces within 40 min. In the study of Biasin et al.,^34^ it was shown that a UV dose of only 3.7 mJ/cm^2^ resulted in >3 log inactivation of the SARS-CoV-2 virus, and complete inactivation was observed with 16.0 mJ/cm^2^. In another study^35^ by the same authors, it was realized that the violet light (405 nm) dose resulting in a 2 log viral inactivation was 10^4^ times less efficient than UV-C (278 nm) light, a plausible explanation for the reduced incidence of the viral infection observed during the summer. By comparing the inactivation efficiency of UVC light on porous and nonporous surfaces contaminated with the SARS-CoV-2 virus, Tomás et al.^36^ realized that higher viral inactivation efficiencies were observed for nonporous surfaces than on porous surfaces. Schuit et al.^37^ examined the virucidal potential of monochromatic UV radiation at 16 wavelengths on the SARS-CoV-2 virus and observed that UVC wavelengths of <280 nm were the most effective. Criscuolo et al.^38^ examined (using ozone and UVC) the decontamination of plastic, glass, gauze, wool, fleece, and wood substrates contaminated with the SARS-CoV-2 virus. Wood proved the most difficult to decontaminate, as a result of its porous structure, thus offering a shelter to virus particles.

The above studies and several others^39,40,49,41−48^ demonstrate the prevalence of UVC methods for decontaminating different material surfaces and the relatively recent application of gaseous ozone for surface sterilization.^33,50−53^ However, a comparative analysis of their performance for the material substrates considered in this study under controlled conditions has hardly been reported in the literature. Furthermore, the application of these decontamination methods for the treatment of porous and nonporous surfaces contaminated with *A. fumigatus* has not been adequately studied. In addition, it is not clear in the published literature if the method of contamination of wet nonporous substrates (droplets versus a film) affects the efficiency of the treatment method. Although several studies have cited the difficulty of achieving adequate UVC penetration in thick substrates or substrates with complex geometry, a detailed quantitative examination of this penetrative limitation is lacking. These knowledge gaps are addressed in the present study and constitute the elements of novelty of this work. We further demonstrate that the peculiar benefits of each method for disinfecting surfaces contaminated with a variety of difficult-to-inactivate microorganisms can be simultaneously explored/combined for improved disinfection efficiency in industrial operations. We present our findings based on a nominal ozone concentration of 10 ppm and UV intensities between 0.26 and 15.56 mW/cm^2^, for exposure durations of 5, 10, and 15 min; the presented findings are thus specific to these conditions. However, these values fall within those, which are popularly utilized in past experimental literature, and are readily attainable under large-scale deployments of these decontamination methods; thus implying their potential wide-ranging applicability.^54^ The applied ozone doses (concentration × time) are between 50 and 150 ppm·min, whereas the UV doses range between 78 and 14,000 mJ/cm^2^ and are further provided in Table S1 (see Supporting Information).

## 2 Methodology

### 2.1 Substrate and Microorganism Preparation

**Figure 1:**
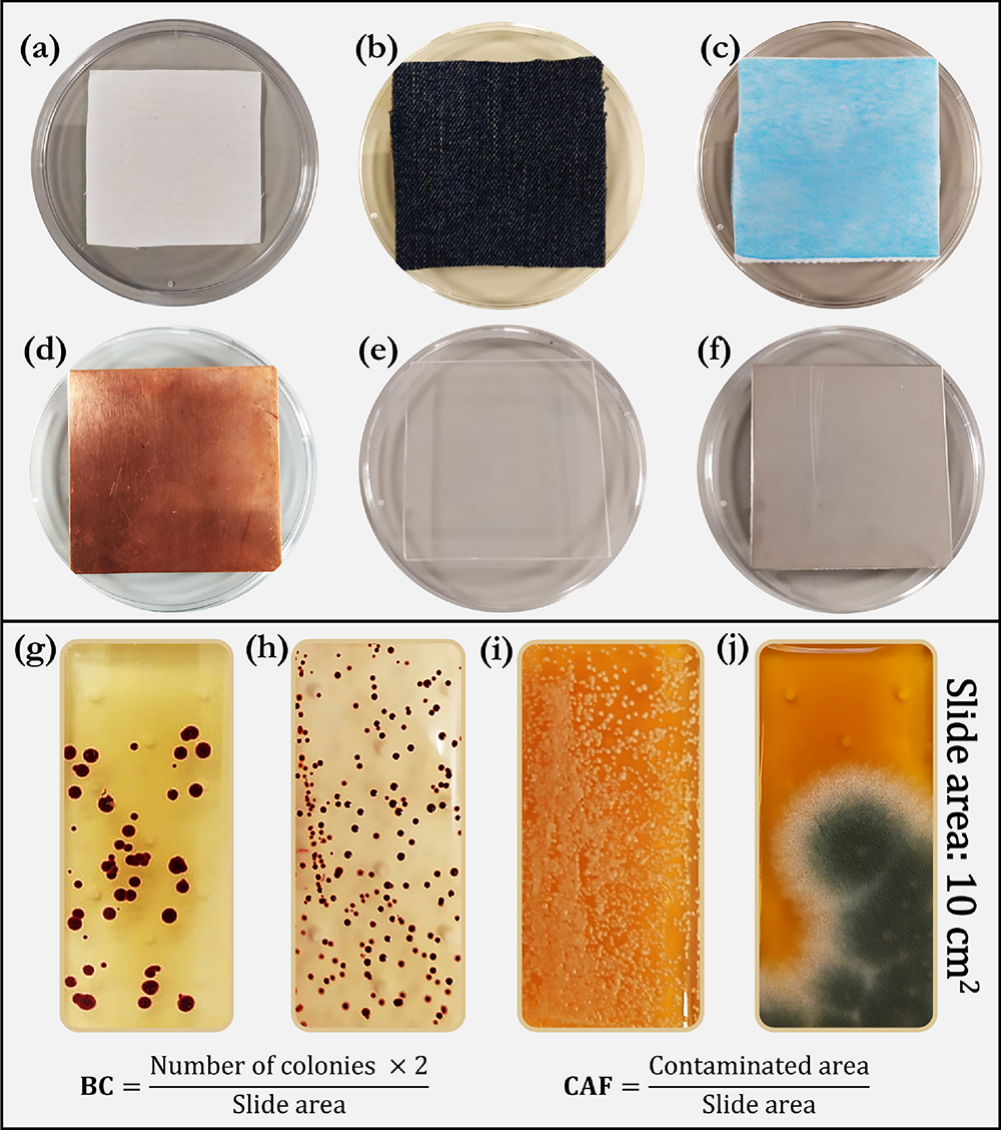
Material substrates utilized for disinfection: (a) 35% cotton–65% polyester swatch, (b) denim swatch, (c) surgical facemask, (d) copper plate, (e) PMMA plate, and (f) stainless steel plate; organisms on the dipslides utilized in this study: (g) *E. coli*, (h) *S. aureus*, (i) *C. albicans*, and (j) *A. fumigatus.* The surface bacterial concentration (BC) was obtained by enumerating the number of colony-forming units on the slide, whereas the fungal contamination on the surface was evaluated by computing the contaminated area fraction on the slide.

The textile substrates utilized in this study are shown in Figure 1a–c, whereas Figure 1d–f illustrates the hard surfaces of copper, PMMA, and stainless steel, with dimensions of approximately 7 cm by 7 cm. Before contamination with the respective organisms, the substrates were sterilized in an autoclave for 20 min at 121 °C and left to dry. Bacterial suspensions utilized for inoculating (100 μL) the substrates were prepared according to the procedure described in Epelle et al.^16,51^

A representative colony of the bacteria (*E. coli* and *S. aureus*) was transferred into 10 mL of nutrient broth (Sigma Aldrich, St. Louis, USA), after which they were incubated in a shaker at 37°C and 150 rpm for 14 h. This was followed by centrifugation of 1 mL of the suspension in a microcentrifuge tube at 10,000 rpm for 5 min.^50^ Washing of the harvested cells with 0.01 M phosphate buffer saline (PBS) solution was performed next, and the suspension’s absorbance (at 570 nm) was adjusted to an optical density (OD) of 0.2 (±0.02), corresponding to 10^9^*E. coli* or *S. aureus* bacteria cells/mL. For the preparation of the fungal inoculum, a 1 cm by 1 cm section of the fungus grown on ISP2 agar (International Streptomyces Project-2 Medium) was obtained and inoculated into sterile ISP2 broth (100 mL). This was followed by shaking at room temperature for 48 h; this allowed for uniform growth of the respective species in their suspensions and subsequent inoculation of the different substrates applied. As shown in Figure 1i,j, the analysis of fungal contamination was carried out by computing the area fractions on the dipslides. Thus, the determination/adjustment of the OD was not deemed necessary.

The analysis of contamination levels (pre- and post-treatment) was achieved via the application of dipslides.^55^ For evaluating the level of bacterial contamination, a nutrient TTC (triphenyltetrazolium chloride) agar slide was applied (Figure 1g,h), whereas fungal contamination was assessed using a malt extract agar slide (Figure 1i,j) in this study. The slides were gently pressed onto the material’s surface for 10 s, after which they were incubated at 37°C for 24–72 h. High-resolution images of the incubated dipslide were subsequently captured and post-processed using the *Colour Thresholder* and *Image Region Analyser* toolboxes of MATLAB (R2020b) (see Figure S2, Supporting Information). Where distinct colonies could be counted, the bacterial concentration (BC) was evaluated by computing the number of colony-forming units per unit area (cm^2^) of the agar slide. However, in the case of severely clustered and mold-like growth, as observed with the fungi, the contaminated area fraction was computed. To evaluate the effectiveness of the treatment procedure, the percentage difference and log reduction values were evaluated. The dipslide method was validated against the conventional Miles and Misra (MM) method (which involves serial dilutions) for counting viable bacteria colony-forming units.

### 2.2 Gas-Phase Ozonation and UVC Disinfection of Contaminated Substrates

**Figure 2:**
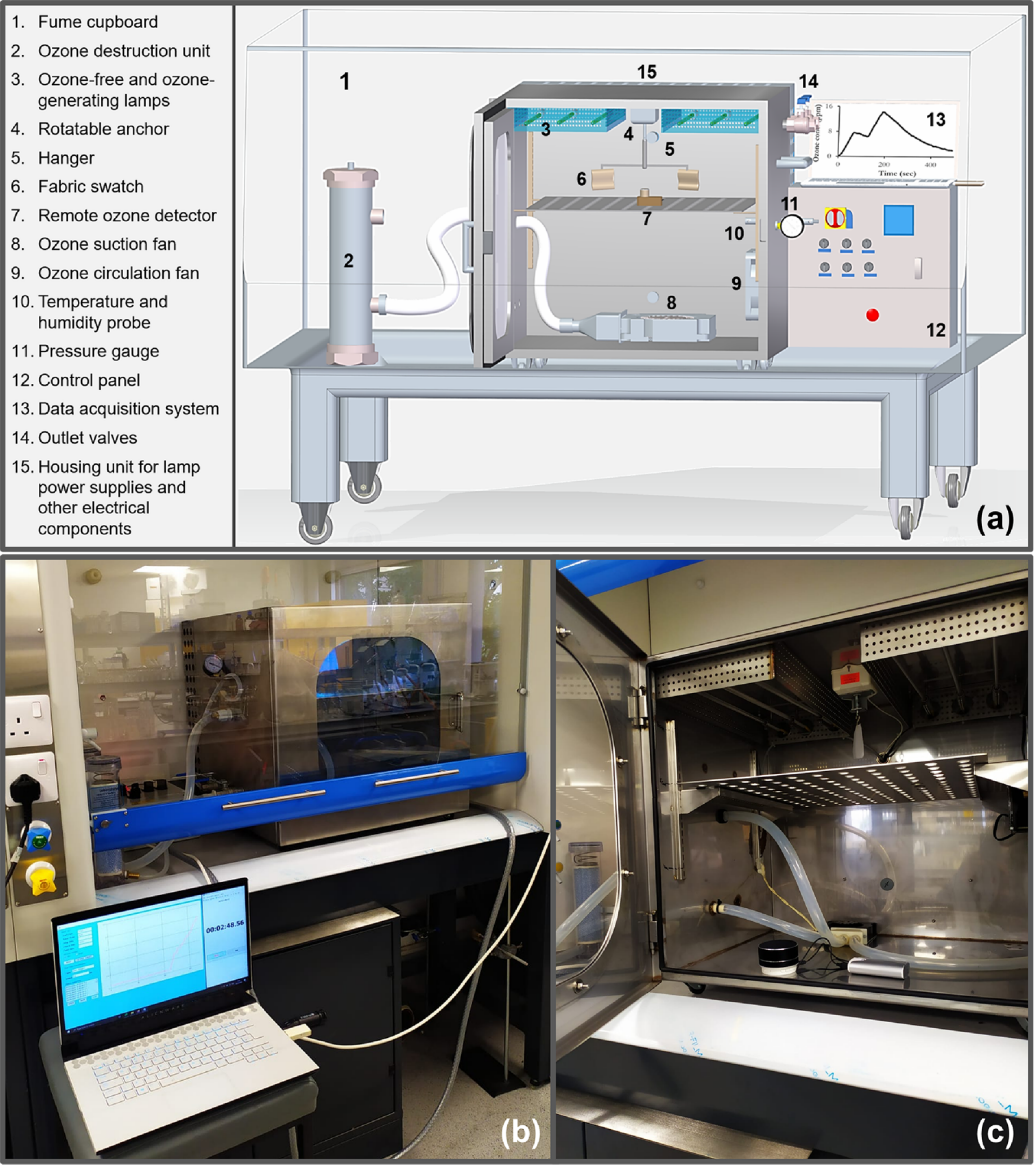
Experimental setup for ozone and UVC disinfection showing (a) a schematic representation and (b) external and (c) internal pictures of the chamber. UV lamps had an operating voltage of 280 V and a total input power of 7 W.

A 3D representation of our bespoke stainless steel sterilization chamber (0.20 m^3^) is shown in Figure 2. As illustrated, ozone generation is achieved by the action of four UV lamps (30 cm in length and 0.9 cm in diameter and a curved surface area of ∼85 cm^2^, Jelight Company Inc. USA), which are an effective source of the 185 nm spectral line, as well as the 254 nm line. Although equipped with multiple inlets for fresh O_2_ supply, we utilize air as the ozone generation medium in this study. The air trapped in the chamber, upon shutting it, was sufficient for the rapid generation of ozone to the desired levels applied for disinfection in this study. The chamber is also fitted with two UVC lamps (mainly 254 nm), which contain a doped quartz envelope, responsible for the absorption of ozone-producing 185 nm photons. This allowed for the evaluation of UVC disinfection alone, in the same chamber. Thus, the only difference between the spectrograms of the ozone-generating (OG) UV lamps and the ozone free (OF) UVC lamps is the absence of the 185 nm spectral line (see Figure S1 of the Supporting Information). The UVC lamps used in this study are of high efficiency, with surface temperatures of only ∼45 °C at peak performance (as per manufacturer specifications). The characteristics of the UV lamps as determined by UV spectral radiometers (Jelight Co Inc, JEL2400) and UVC detectors (Jelight Co Inc, JXSD140T254) are provided in the Supporting Information (Figure S1b–d). The lamps have an operating period of 30,000 h, and less than 0.1% of this time has been spent.

The adjustable shelf was utilized to accurately alter the distance of the substrate from the light source. In the provided 3D representation of the apparatus (Figure 2), there is a perforated stainless steel plate attached to the adjustable shelf/platform that allows accurate variations in the distance (from the lamps) to be implemented. Positions that corresponded to the required distances were marked and labeled in the chamber. During the UVC disinfection experiments, the substrates were placed flat on a disc and mounted on the shelf, after which they were irradiated with 1 UVC lamp for the desired duration. Repeatability in the distance was readily attained with this procedure. As will be shown in the results section, this distance affects the UV intensity and is critical to the disinfection efficiency of the UVC method.

For efficient ozone exposure, the substrates are supported using the hanger attached to a rotating device (2.5 rpm). The axial and centrifugal fans ensure efficient circulation and rapid removal of ozone gas in and from the chamber. The catalytic destruct unit facilitates the breakdown of ozone to oxygen in a fume cupboard, equipped with an effective extraction system. The internal components of the chamber are all coupled to a control panel, whereas the readings of ozone concentration, temperature, and humidity are collected via a data acquisition software on a computer; the average temperature and relative humidity in the chamber were 20 °C and 40%, respectively. The ozone concentration is measured using two methods, a probe attached to a monitor (Bosean Ltd. China) and via remote sensing of the gas (WinSensors Ltd. China), which connects to the data acquisition system via Bluetooth. While the position of the probe was fixed, the remote sensor could be positioned at any location in the chamber. However, based on numerous preliminary tests conducted, the position of the sensor did not matter because of the efficient gas circulation in the chamber, thus yielding homogeneous gas distribution and concentration. When the fan was off, it took longer than usual for ozone to be picked by the sensor, thus demonstrating the key influence of efficient circulation in the system. Repeatable ozone concentration measurements were obtained as judged by a standard deviation of <0.2 ppm. A control panel (Belmos Electrical Services, UK) couples all electrical components within the chamber, allowing for a systematic variation of different parameters affecting the system (such as the number of lamps switched on, the suction rate of the centrifugal fan, and the rotation of the porous substrates).

The response time of the sensor, the rapid auto-decomposition of ozone, and the inherent mechanism of UV-based ozone generation implied that the lamps had to be turned off at specific times to avoid concentrations higher than desired and turned on to avoid lower concentrations. To maintain the ambient ozone concentration at the desired levels for the required duration, a carefully planned on and off sequence was implemented. A better/less laborious approach would have been to automate this process; however, our manual-type control did not affect the accuracy. Ozone treatments of the substrates were either carried out at distances of either 30 (hanging with rotation) or 60 cm (flat, at the base of the chamber) away from the UV light source; these distances and orientations implied very minimal UVC exposure. Furthermore, the generation rate with three lamps (Figure S1, Supporting Information), shows that the desired ozone concentration (10 ppm) can be readily obtained in less than 2 min. With the four ozone generation lamps utilized in this study, we were able to attain 10 ppm in less than a minute of operation, after which the lamps are turned off. This rapid generation time further mitigates UVC exposure during ozone treatment. The manual control (on/off sequence applied to counter ozone’s auto-decomposition), only involved turning on the lamps for as little as 8 s to raise the concentration again to the desired levels; this happened only 3 times in a 15 min cycle. It is worth mentioning that the total exclusion of other reactive species/interferences when applying advanced oxidative processes for decontamination applications is extremely difficult. The use of corona discharge for ozone generation would have produced a more significant interference (e.g. nitrogen- and oxygen-based species), particularly when utilizing air as the precursor gas. Furthermore, channeling ozone gas through a duct into the chamber would have resulted in the pressurization of the chamber (a safety hazard) and a consequent need for depressurization, which also makes it more difficult to consistently control the desired ozone concentration and ensure the repeatability of results. Our implemented UV generation method achieves excellent repeatability in the ozone generation profiles.

Besides evaluating the impact of UVC intensities (at different distances from the light source) on the disinfection efficiency, the combined effects of ozone and UVC were also analyzed. Since UVC destroys ozone as demonstrated in Figure S1 (Supporting Information), it was impractical to simultaneously expose the contaminated materials to ozone and UVC as this would have made it difficult to accurately maintain the ozone concentration at the desired level in the chamber. Rather, a sequential treatment procedure was utilized. Thus, a 5 min combined (O_3_ + UVC) treatment implies an ozone treatment for 2.5 min, followed by UV treatment for 2.5 min (the ozone concentration was brought to 0 before UVC exposure. Furthermore, the effect of UVC penetration on the disinfection efficiency of the porous substrate (cotton-polyester fabric swatch) was also analyzed. This was achieved by utilizing a sterile barrier consisting of 1–7 layers of swatches between an upper and lower contaminated swatch. Furthermore, the effect of different substrate types, contamination method (a droplet or a smeared film on the nonabsorbent surface), and the impact of air circulation on UVC disinfection are analyzed. It is worth mentioning that the bacterial and fungal suspensions were not dried onto the surface of the material substrate; the treatments were performed in their wet conditions.

### 2.3 Scanning Electron Microscopy (SEM)

The preparation of the samples for SEM began by transferring 50 μL of the microbial suspension onto the substrate of interest (already mounted on aluminum stubs via conductive double-sided carbon tape) and incubating this at 37°C for 4 h. Washing with 0.01 M PBS followed, after which fixation at room temperature was performed with 2% glutaraldehyde, 2% paraformaldehyde, 0.1M phosphate buffer solution for 30 min. Sequential dehydration of the samples was achieved by adding 50, 70, 80, 90, 95, and 99% ethanol *v*/*v*, with intermittent rinsing for 2 min occurring between each dehydration step. Freeze drying after *tert*-butanol treatment was subsequently performed. Gold sputter coating (∼10 nm) using a coater (Emscope SC500) followed, after which samples were viewed on a Hitachi S-4100 SEM, running at 5–10 kV.

## 3 Results and Discussion

### 3.1 Effect of Distance from the Light Source

**Figure 3:**
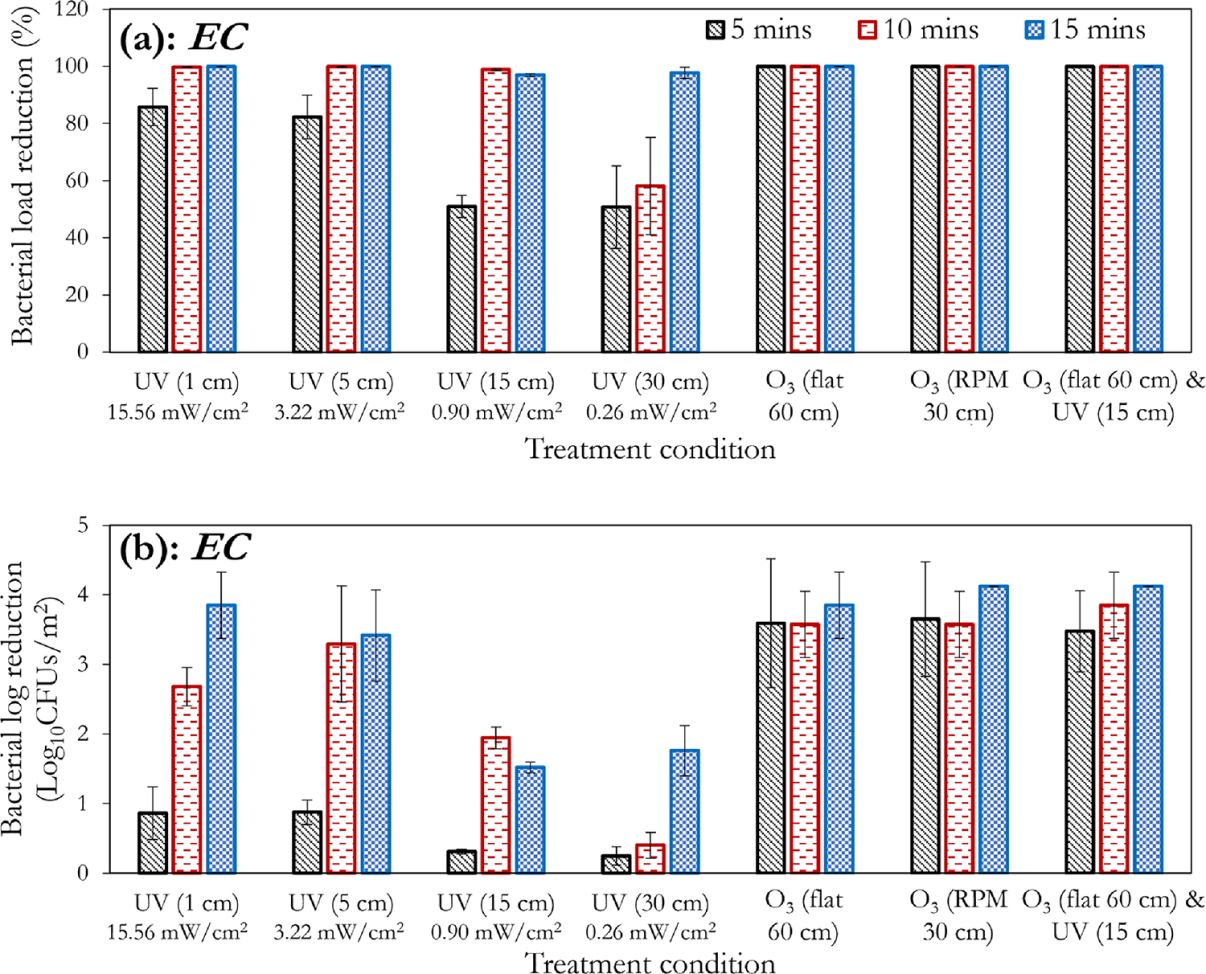
Effect of treatment duration, distance between lamp and substrate or UVC intensity, and substrate orientation for UVC and ozone (10 ppm) treatment of cotton-polyester fabric swatches contaminated with *E. coli* showing the (a) percentage and (b) log reductions. Error bars represent the standard deviations of three separate runs.

**Figure 4:**
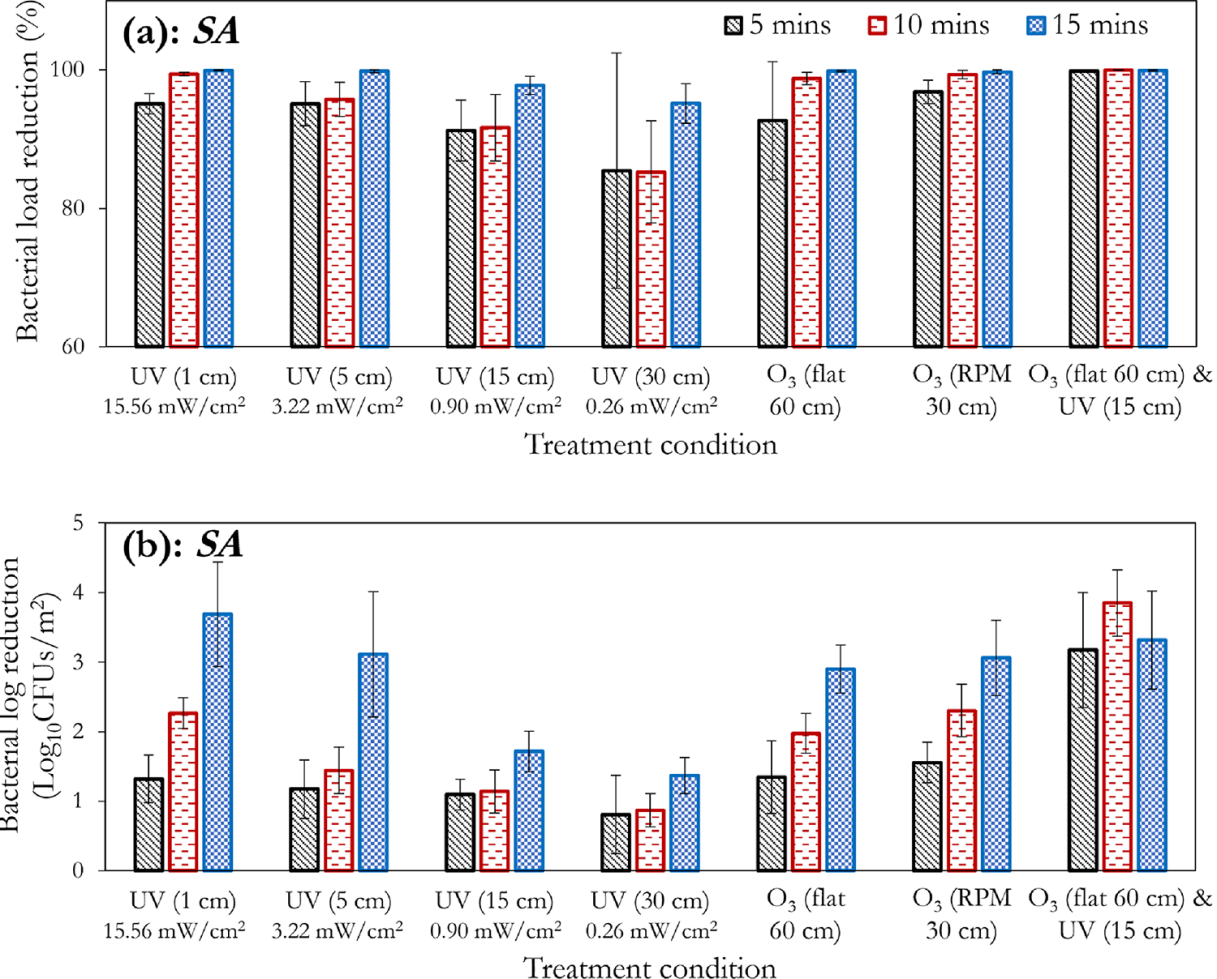
Effect of treatment duration, distance between lamp and substrate or UVC intensity, and substrate orientation for UVC and ozone (10 ppm) treatment of cotton-polyester fabric swatches contaminated with *S. aureus* showing the (a) percentage and (b) log reductions. Error bars represent the standard deviations of three separate runs.

**Figure 5:**
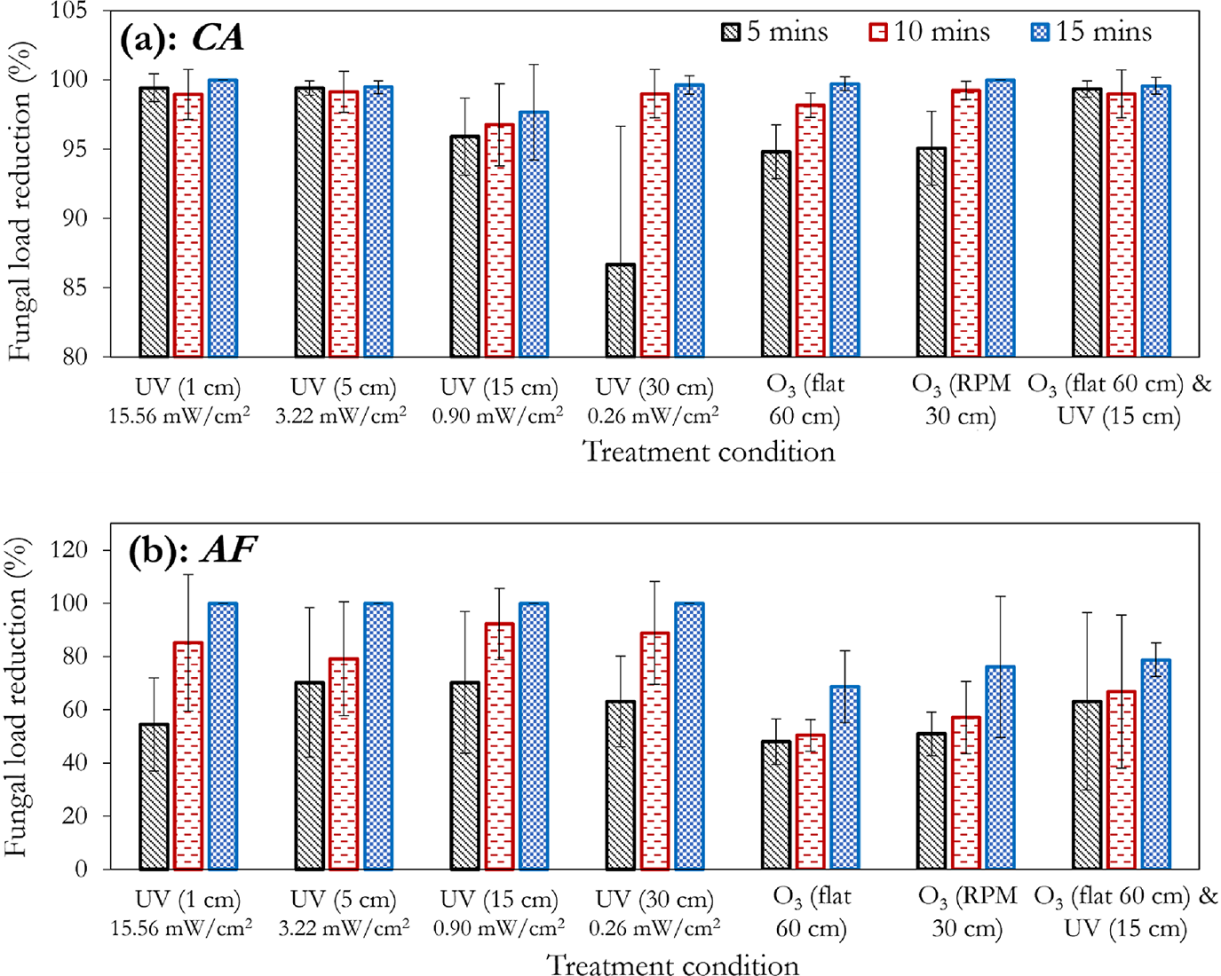
Effect of treatment duration, distance between lamp and substrate or UVC intensity, and substrate orientation for UVC and ozone (10 ppm) treatment of cotton-polyester fabric swatches contaminated with (a) *C. albicans* and (b) *A. fumigatus*. Error bars represent the standard deviations of three separate runs.

The disinfection efficiencies of UVC treatment at different distances/UV doses (1, 5, 15, and 30 cm, Table S1) between the lamps and the contaminated substrate are shown in Figures 3–5 for different treatment durations. These distances correspond to UVC intensities of 15.56, 3.22, 0.90, and 0.26 mW/cm^2^, respectively. Furthermore, Figure 3 captures the effect of ozone treatment when the substrate is left flat (at the base of the chamber) and when the substrate is vertically attached to the rotating anchor; the combined effect of ozone and UVC treatment is also shown. It is worth mentioning that a nominal ozone concentration of 10 ppm has been applied, and the substrate utilized here is the cotton-polyester fabric swatch as shown in Figure 1a.

As the distance from the UVC light source increases, the disinfection efficiency reduces as observed in Figures 3a and 4a and more evidently in the bacterial log reduction plots (Figures 3b and 4b). However, this decrease in disinfection efficiency does not tend to follow the exponential decay of the intensity as shown in Figure S1 (see Supporting Information). Additionally, it is observed that a reasonable disinfection efficiency (>1.5 bacterial log reduction) can be achieved at the 30 cm distance (0.26 mW/cm^2^), provided that the exposure time is up to 15 min. However, a 1 cm distance (15.56 mW/cm^2^) provides >4 log reductions for the same treatment duration of 15 min. As with the bacteria, the fungi applied herein (*C. albicans* and *A. fumigatus*) generally showed improvements in the percentage reduction with increasing treatment duration. *A. fumigatus* had a low resistance to UVC treatment; at a 30 cm distance (0.26 mW/cm^2^) from the substrate, it can be observed that it is 100% inactivated at 15 min of exposure (Figure 5b), compared to 97.78 (Figure 3a), 95.12 (Figure 4a), and 99.62% (Figure 5a) for *E. coli*, *S. aureus*, and *C. albicans*, respectively.

For the bacteria, the difference between ozone treatment and UVC can be more readily observed at the 5 and 10 min treatment durations. The enhanced effect of 10 ppm ozone treatment is clearly demonstrated by the higher log reduction values, particularly for *E. coli* (Figure 3b). This may be attributed to the increased penetration of gaseous ozone, irrespective of the substrates’ orientation. This outperformance of ozone relative to UVC treatment was also observed with *C. albicans* (Figure 5a). Conversely, 10 ppm of ozonation was insufficient to match the impact of UVC on *A. fumigatus*, even at the most unfavorable distance of 30 cm (0.26 mW/cm^2^). This demonstrates the greater effect of UVC on *A. fumigatus* compared to 10 ppm ozone treatment. It is worth mentioning that this observation is limited to the applied 10 ppm of ozone concentration, as higher concentrations (20 ppm) have been shown to have a more efficient fungicidal effect on *A. fumigatus* in just 4 min.^50^ Furthermore, at 10 ppm, only marginal improvements in the disinfection efficiency are observed by changing the orientation of the substrate from flat to hanging (with rotation). However, it is worth mentioning that this difference may be amplified, when the substrate is larger, and prone to obstructions, as is usually the case in a real/industrial system. For the disinfection of PPE and other garments, this is likely to be improved when they are adequately hung in a chamber, compared to when they are piled or folded.

Furthermore, the combined effect of UVC and ozone has been examined in a sequential manner in this study, mainly because of the highlighted impact of OF lamps on the OG lamps (Figure S1, Supporting Information). Thus, a 5 min combined treatment involved 2.5 min ozone exposure at 10 ppm followed by immediate UVC treatment for 2.5 min. A simultaneous treatment would have resulted in some interference since ozone is highly absorbing of UVC; this affects the attainment of the desired ozone concentration under the same exposure duration, as well as the UVC intensity. Nonetheless, we envisage that the impact of this interference on the attainable inactivation efficiencies will be minimal. This is because the absorbance effect of ozone on UVC will lead to the further production of free radicals, which in turn contribute to microbial inactivation. The production of these radicals will likely counterbalance this effect of reduced UVC intensity and ozone concentration.

As shown in Figures 3b and 4b, the effect of the combined (sequential) treatment is an overall improvement in the disinfection efficiency compared to the independent application of ozone and UVC (at any distance or intensity) for *E. coli* and *S. aureus.* A similar observation was reported by Magbanua et al.^56^ However, this is not exactly the case with *C. albicans*, as the UVC treatments at 1 (15.56 mW/cm^2^) and 5 cm (3.22 mW/cm^2^) still supersede the combined treatment (Figure 5a). Conversely, comparing the 15 cm (0.90 mW/cm^2^) UVC treatment and that of the combined treatment shows that the independent application is not as effective (Figure 5a). The independent application of UVC, however, still trumps the combined treatment of *A. fumigatus*, confirming the effectiveness of UVC against this pathogen. This combined treatment demonstrates the potential to leverage the peculiar disinfection properties of both methods, without the sole reliance on very high ozone concentrations, or the independent application of long UVC exposure durations, both of which may negatively impact the material/substrate via severe oxidation, fiber degradation, discoloration, or embrittlement.

Our results further demonstrate that prolonged exposure of the substrate to UVC at lower intensities may provide improved inactivation compared to high-intensity exposures for short durations. For example, this can be observed when comparing the 15.56 mW/cm^2^ exposure for 5 min, with the 0.90 mW/cm^2^ exposure for 15 min for *E. coli*, Figure 3b. This is also observed in Figures 4b and 5b. The potential for cell stacking in the growth patterns of the organisms, given the high inoculum concentrations utilized, implies that sufficient time is required for adequate UVC penetration and consequently complete inactivation. Thus, the use of UVC dose as the sole measure for assessing inactivation efficiency may limit the understanding of the requirements for full inactivation; hence our reporting of the full set of conditions (distance, intensity, exposure duration, and dosage).

**Figure 6:**
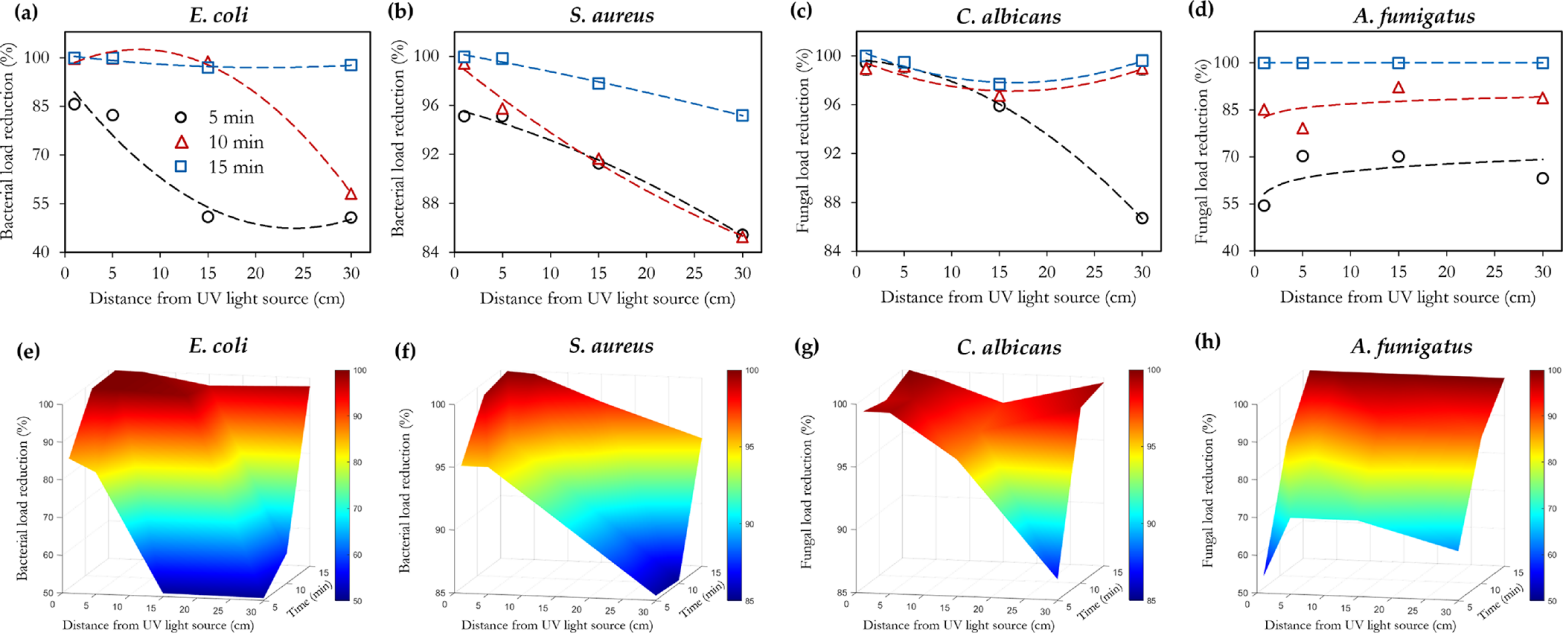
Effect of exposure time, and substrate distance from UVC light source on the disinfection efficiency of UVC. Contour plots (e–h) are generated from the experimental data points (a–d) via Delaunay triangulation for enhanced visualization of these effects. The distances of 1, 5, 15, and 30 cm from the lamp correspond to intensities of 15.56, 3.22, 0.90, and 0.26 mW/cm^2^, respectively.

Figure 6 (obtained by postprocessing the UVC-only sections of Figures 3–5) is presented to better visualize the impact of the distance from the UVC light source on the disinfection efficiency. The greater relative sensitivity of the tested fungi to the bacteria is further portrayed in Figure 6, as indicated by the respective areas of the blue regions (for example *E. coli* vs *A. fumigatus*, and *S. aureus* vs *C. albicans*). A threshold effect (with respect to exposure time) can be observed in Figure 6a (*E. coli*), where it takes an exposure duration of >5 min to see significant improvements in the log reduction. A similar observation using ozone has been reported in the studies by Broadwater et al.^57^ and Kowalski et al.^58^ However, with *S. aureus* (Figure 6b), this threshold appears to be 10 min. For *C. albicans*, this threshold time (5 min) is observed to exist only at the 30 cm mark (0.26 mW/cm^2^), whereas *A. fumigatus* did not strongly demonstrate this threshold effect.

**Figure 7:**
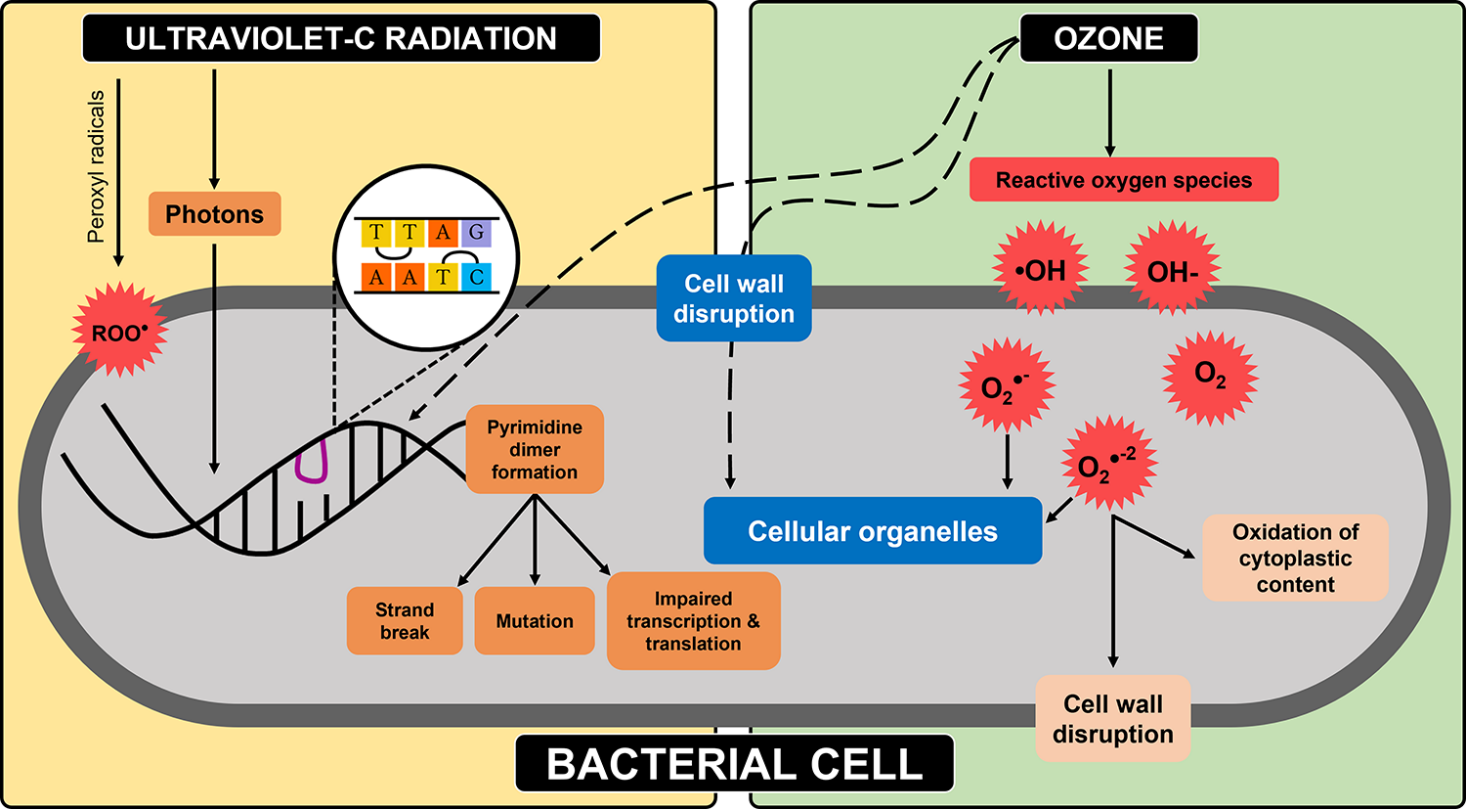
Mechanism of ozone and UVC inactivation of a bacterial cell.

In summary, while UVC mainly attacks the cell DNA, ozone, and the many free radicals it generates, it oxidizes several cell constituents (including the membrane, DNA, cytoplasmic contents, and other organelles). Thus, the combined action of ozone and UV results in simultaneous interference on several aspects of the cell’s metabolism, leading to its more rapid inactivation in a majority of the cases, compared to the independent application of ozone and UVC treatments. Furthermore, we hypothesize that the thicker and tougher cell wall of the fungal species relative to the bacteria makes it more difficult for gaseous ozone to penetrate the fungal cell (and eventually oxidize the cells’ constituents) compared to the high penetrative efficiency of UV photons (which attack the cell’s DNA according to Figure 7). However, in the case of bacteria, the multifaceted effects of ozone on different cell constituents (DNA, cell wall, cytoplasm, etc.) and the lower penetrative resistance posed by the cell wall make ozone more potent on the bacteria (particularly *E. coli*), as shown by the results in Figures 3–5. Furthermore, bacteria applied in this study typically have a cross-linked polymer peptidoglycan amorphous structure, whereas the fungi mainly have a linear and more crystalline polymer chitin structure. We further hypothesize that this difference in structure determines the effectiveness of UVC radiation. Thus, the significantly crystalline structure of the fungi cell wall enables better utilization of the incident UVC radiation for its inactivation compared to the amorphous cell wall structure of the bacteria. However, further work is required to elucidate the impact of these cell wall structures on UVC radiation. It is also worth mentioning that the UVC dosage requirements reported for effective inactivation of SARS-CoV-2 (>99.9%) in the work of Biasin et al.^34^ were in the range of 3–16 mJ/cm^2^. However, our study demonstrates that up to 234 mJ/cm^2^ (Table S1, Supporting Information) is required for complete inactivation of *A. fumigatus*. This significant difference demonstrates the importance of evaluating the specific sensitivities of the organisms of interest, before administering the required UVC dosage, for effective decontamination. Another important observation that substantiates this point is the fact that UVC was observed to outperform ozone for all tested conditions in the work of Criscuolo et al.^38^ However, our study illustrates that this outperformance significantly depends on the organism; the tested fungi showed more sensitivity to UVC treatments than the bacteria.

### 3.2 Analysis of UVC and Ozone Penetration

**Figure 8:**
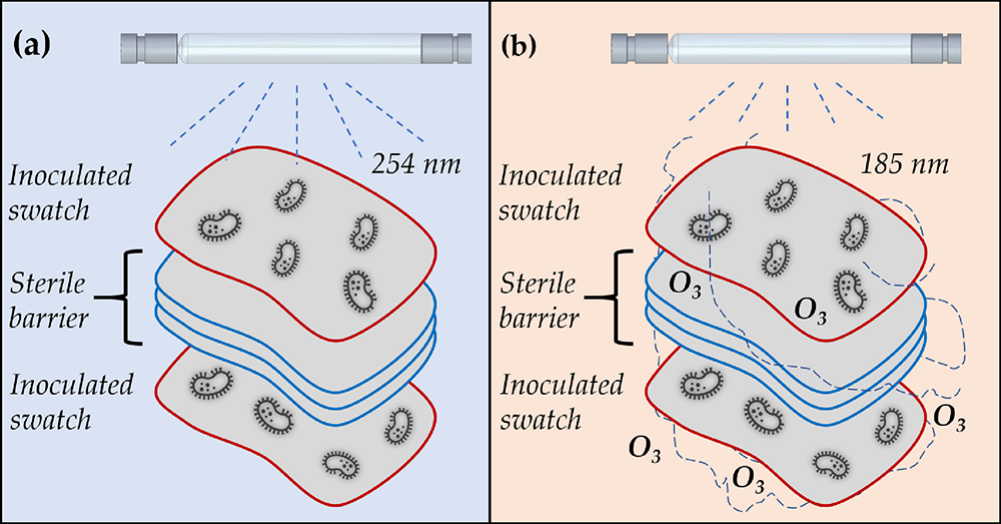
A set of stacked cotton-polyester fabric swatches utilized to study the effect of (a) UVC and (b) ozone penetration on the disinfection efficiency of the tested organisms. Each swatch is 0.5 mm thick.

To analyze the penetration of UVC and ozone, for microbial disinfection, the stacked set of cotton-polyester fabric swatches shown in Figure 8 was utilized (placed on a Petri dish and inserted in the chamber). The thickness of the sterile barrier was altered between 1 and 7 layers to evaluate the extent of penetration using all four organisms. For this penetration study, the fabric swatches are irradiated at a UV intensity of 15.56 mW/cm^2^, corresponding to a distance of 1 cm from the lamp (i.e., the distance from the topmost swatch). As observed in Table 1 (or Figure S3a–d of the Supporting Information), ozone outperforms UVC treatment for top-positioned fabric swatches contaminated with *E. coli* and *S. aureus*; however, the reverse was the case with the fungi (Table 1 or Figure S3e,f, Supporting Information). While there was a marginal improvement (by UVC over ozone) for the inactivation of *C. albicans*, the disparity between both treatment methods is more significant with *A. fumigatus* for the top-positioned contaminated swatch. For the lower-positioned swatches, UVC treatment was unable to penetrate a single layer barrier for efficient bacterial inactivation (<1 log reduction ) as observed in Table 1 or Figure S3b,d of the Supporting Information. This was also the case for *A. fumigatus*; however, effective inactivation of *C. albicans* was recorded with one sterile barrier (Table 1 or Figure S3e, Supporting Information) by UVC treatment. The similarity in the inactivation of the bacteria on the lowermost swatch (although minimal) by UVC despite the difference in the number of sterile layers may be attributed to the minimally reflected^59^ UVC rays by the stainless steel walls of the chamber (especially as the two UVC lamps are located closest to the chamber walls on either side.

**Table 1:** Effect of the Number of Sterile Layers on the Penetration of UVC (1 cm or 15.56 mW/cm^2^ and 15 min) and Ozone (10 ppm, 15 min) for Microbial Inactivation^a^

		UVC				ozone			
organism	position of contaminated swatch (treatment method & number of sterile layers)	1	3	5	7	1	3	5	7
EC	uppermost (PR & LR)	99.796% ± 0.28	99.900% ± 0	99.875% ± 0.11	100% ± 0	100% ± 0	100% ± 0	100% ± 0	100% ± 0
		3.26 ± 1.22	3.00 ± 0	3.00 ± 0.43	4.12 ± 0	4.12 ± 0	4.12 ± 0	4.12 ± 0	4.12 ± 0
	lowermost (PR & LR)	46.250% ± 1.77	43.750% ± 1.78	46.250% ± 5.30	42.500% ± 0	99.050% ± 0.78	98.075% ± 0.81	98.375% ± 0.39	98.050% ± 0.28
		0.27 ± 0.01	0.25 ± 0.01	0.27 ± 0.04	0.24 ± 0	2.11 ± 0.41	1.74 ± 0.19	1.80 ± 0.10	1.71 ± 0.06
SA	uppermost (PR & LR)	99.875% ± 0.04	99.888% ± 0.02	99.850% ± 0	99.85% ± 0	99.900% ± 0.07	99.888% ± 0.05	99.900 ± 0.07	99.913% ± 0.05
		2.91 ± 0.13	2.95 ± 0.07	2.82 ± 0	2.82 ± 0	3.06 ± 0.34	2.97 ± 0.21	3.06 ± 0.34	3.10 ± 0.28
	lowermost (PR & LR)	24.375% ± 0.88	22.375% ± 0.18	24.875% ± 0.53	24% ± 1.06	97.400% ± 2.12	97.075% ± 1.45	97.325 % ± 1.31	97.150% ± 0.99
		0.12 ± 0.01	0.11 ± 0	0.12 ± 0	0.12 ± 0.01	1.67 ± 0.40	1.56 ± 0.23	1.60 ± 0.22	1.56 ± 0.15
CA	uppermost (PR)	100% ± 0	100% ± 0	100% ± 0	100% ± 0	100% ± 0	99.643% ± 0.51	99.286% ± 0	99.643% ± 0.51
	lowermost (PR)	98.500% ± 0.10	40% ± 16.16	40.143% ± 4.24	42.857% ± 10.10	97.130% ± 0.97	97.150% ± 1	97.200% ± 1.07	97.129% ± 0.97
AF	uppermost (PR)	100% ± 0	100% ± 0	100% ± 0	100% ± 0	51.425% ± 2.51	52.520% ± 0.48	52.960% ± 0.27	49.955% ± 0.93
	lowermost (PR)	27.730 ± 7.31	26.160 ± 13.35	37.135 ± 5.95	30.925 ± 6.89	31.630% ± 0.45	32.885% ± 3.46	44.170% ±21.88	37.895% ± 4.52

a Standard deviations are obtained from three separate runs. EC, *E. coli*; SA, *S. aureus*; CA, *C. albicans*; AF, *A. fumigatus*; PR, percentage reduction; LR, log reduction. Equivalent plots of the data in this table are presented in the Supporting Information (Figure S3).

The best results of UVC inactivation constrained by penetration barriers were achieved with *C. albicans*; this may be attributable to the mechanisms proposed in Figure 7; however, a more thorough analysis at a cellular level is required to elucidate the fungi sensitivities to UVC. The inherent advantage of ozone penetration in the gaseous phase is evident at the lowermost layers, achieving far higher bacterial reductions compared to UVC treatments. In the case of *S. aureus* (Table 1 or Figure S3c, Supporting Information), the percentage reduction achieved for UVC treatment is quadrupled by ozone’s application, whereas with *E. coli* (Table 1 or Figure S3b, Supporting Information) and *C. albicans* (Table 1 or Figure S3e, Supporting Information), it is correspondingly doubled. Although ozone still outperformed UVC in the treatment of *A. fumigatus* (for the bottommost swatches), the difference is relatively mild (Table 1 or Figure S3f, Supporting Information) compared to the other three organisms. As demonstrated in the work of Epelle et al.,^50^ up to 20 ppm of ozone concentration is required for fungicidal effects to be observed with *A. fumigatus*. For certain large-scale applications, where this concertation is difficult to attain, UVC treatment can be combined for increased effectiveness against this fungus.

### 3.3 Effect of Material Type on Disinfection Efficiency

The antimicrobial efficacies of ozone and UVC against the applied bacteria, on the different materials utilized in this study, are shown in Table 2 or Figure S4 (Supporting Information). It is worth mentioning that the same inoculum volume (100 μL) of the bacterial and fungal suspensions was utilized in all scenarios. While the porous materials (cotton-polyester and denim) readily absorbed the suspension once inoculated, the contamination of the water-repellent surgical mask and hard surfaces (copper, stainless steel, and PMMA) involved the application of droplets (10 by 10 μL of the suspension) onto the surfaces. An equivalent performance (100% bacterial inactivation or >4 log reduction) by both treatment methods is observed on all hard substrates (Table 2 or Figure S4, Supporting Information). The recoveries from the control samples for all nonabsorbent material substrates were the same; this is particularly because the samples were treated in their wet states. Nonetheless, this similarity of inactivation efficacies (particularly on the nonadsorbent materials) is also consistent with the findings of Hudson et al.^33^ and Tizaoui et al.^52^ These studies featured the drying of the contaminant on the surface of the material substrate. Thus, it can be concluded that both wet and dry substrates are equally disinfected by UVC and ozone. We also attribute this similarity of results shown in Table 2 (for the nonabsorbent materials), to the intensity of the ozone and UV treatments utilized. In essence, we utilized the best conditions (highest UV intensity and longest exposure duration) to determine if the material type had an impact under these conditions. Our observations invariably show that these treatment conditions overshadow any possible contributions to antimicrobial activity provided by the material type. For the textile substrates, denim proved the most difficult to sterilize. This can be attributed to the thick fibers and multilayered structure of the denim swatch, posing a challenge for UVC and ozone penetration. UVC can also be seen to outperform ozone for the disinfection of the denim material against both bacteria. Although the penetration efficiency of ozone was demonstrated to be superior over multiple stacked porous cotton-polyester swatches (Table 1 or Figure S3, Supporting Information), the penetration capability of UVC may be better experienced over singular thick pieces of porous clothing. This may be attributable to the tightly packed weave architecture of the denim material relative to the more porous cotton-polyester material. While the penetrative power of ozone is clearly established (Table 1), certain weave architectures of textiles may be better disinfected by UVC, again demonstrating the complementary performances of both methods.

**Table 2:** Effect of Material Type on the Disinfection Efficacy of Ozone (10 ppm 15 min) and UVC (1 cm or 15.56 mW/cm^2^ and 15 min) Treatments for the Different Bacteria and Fungi^a^

		UV					
organism	contamination method (treatment method/material type)	C/P	denim	FM	Cu	SS	PMMA
EC	10 μL droplet (PR & LR)	100% ± 0	98.900% ± 0.92	99.871% ± 0.17	100% ± 0	100% ± 0	100% ± 0
		4.12 ± 0	2.05 ± 0.42	3.36 ±1.08	4.12 ± 0	4.12 ± 0	4.12 ± 0
	full smear (PR & LR)			100% ± 0	100% ± 0	100% ± 0	100% ± 0
				4.12 ± 0	4.12 ± 0	4.12 ± 0	4.12 ± 0
SA	10 μL droplet (PR & LR)	100% ± 0	99.900% ± 0	100% ± 0	100% ± 0	100% ± 0	100% ± 0
		4.12 ± 0	3.00 ± 0	4.12 ± 0	4.12 ± 0	4.12 ± 0	4.12 ± 0
	full smear (PR & LR)			100% ± 0	100% ± 0	100% ± 0	100% ± 0
				4.12 ± 0	4.12 ± 0	4.12 ± 0	4.12 ± 0
CA	10 μL droplet (PR)	100% ± 0	100% ± 0	100% ± 0	99.83% ± 0.2	100% ± 0	100% ± 0
	full smear (PR)			100% ± 0	100% ± 0	100% ± 0	100% ± 0
AF	10 μL droplet (PR)	100% ± 0	100% ± 0	12.75% ± 1.06	5.05% ± 7	6.340% ± 1.61	9% ± 12.59
	full smear (PR)			93.285% ± 4.01	72.035% ± 18.83	89.855% ± 14.35	92.155% ± 2.57

		ozone					
organism	contamination method (treatment method/material type)	C/P	denim	FM	Cu	SS	PMMA
EC	10 μL droplet (PR & LR)	100% ± 0	94.375% ± 2.93	100% ± 0	100% ± 0	100% ± 0	100% ± 0
		4.12 ± 0	1.28 ± 0.24	4.12 ± 0	4.12 ± 0	4.12 ± 0	4.12 ± 0
	full smear (PR & LR)			100% ± 0	100% ± 0	100% ± 0	100% ± 0
				4.12 ± 0	4.12 ± 0	4.12 ± 0	4.12 ± 0
SA	10 μL droplet (PR & LR)	100% ± 0	99.590% ± 0.18	100% ± 0	100% ± 0	100% ± 0	100% ± 0
		4.12 ± 0	2.43 ± 0.19	4.12 ± 0	4.12 ± 0	4.12 ± 0	4.12 ± 0
	full smear (PR & LR)			100% ± 0	100% ± 0	100% ± 0	100% ± 0
				4.12 ± 0	4.12 ± 0	4.12 ± 0	4.12 ± 0
CA	10 μL droplet (PR)	100% ± 0	95.784% ± 1.78	60.137% ± 1.35	36.121% ± 4.49	34.421% ± 1.47	28.516 ± 14.40
	full smear (PR)			99.316% ± 0.22	100% ± 0	100% ± 0	100% ± 0
AF	10 μL droplet (PR)	60.220% ± 21.21	66.500% ± 3.54	1.500% ± 0.02	2% ± 0	1.500% ± 0.0	1.500% ± 0.0
	full smear (PR)			5.315% ± 5.40	4.730% ± 2.02	2% ± 1.41	14.545% ± 9.36

a Standard deviations are obtained from three separate runs. EC, *E. coli*; SA, *S. aureus*; CA, *C. albicans*; AF, *A. fumigatus*; PR, percentage reduction; LR, log reduction; C/P, cotton-polyester fabric swatch; FM, face mask; Cu, copper; SS, stainless steel; PMMA, poly(methyl methacrylate). The smear tests were performed on the nonporous substrates only and hence the empty fields for C/P and denim, which are porous. Equivalent plots of the data in this table are presented in the Supporting Information (Figures S4 and S5).

In this section of material disinfection, a distinction between the bacterial (Table 2 or Figure S4, Supporting Information) and fungal (Table 2 or Figure S5, Supporting Information) inactivation efficiencies has been made because of a rather peculiar behavior observed with the fungus. This pertains to the marked dependence of the disinfection efficiencies attained on the method of contamination (via droplets on the surface or a smeared film). Table 2 (or Figure S5a, Supporting Information) comparatively illustrates ozone and UVC performance for disinfecting materials contaminated via 10 by 10 μL of the *C. albicans* suspension. Since the suspension is readily absorbed by the fibers of the fabric swatch, the method of contamination is inconsequential, and the results of the cotton-polyester and denim swatches demonstrated effective inactivation by ozone and UVC. However, as previously observed with the bacteria in Table 2 and Figure S4, UVC outperforms ozone for denim decontamination (Table 2 or Figure S5a), whereas the highly porous cotton-polyester swatch shows no difference in the decontamination efficiency obtained by ozone and UV treatments. On the nonabsorbent surfaces, 10 ppm ozonation is not sufficient to decontaminate the surfaces, harboring the droplets of the *C. albicans* suspension. On changing the method of contamination to a film on the surface of the nonabsorbent materials, it can be observed that the performance of ozone significantly increases to match that of UVC (Table 2 or Figure S5b). This increased contact area for ozone penetration is a likely reason for this observation with *C. albicans*. Although copper is known to have antimicrobial properties, the required killing time reported in the literature for *C. albicans* is roughly 24 and >120 h for *A. fumigatus*.^60,61^ The exposure durations applied herein before ozone and UVC treatments are significantly shorter (∼2min) and may be attributed to the absence of this antimicrobial property relative to other materials in this study. According to Table 2 or Figure S5c (*A. fumigatus*), the porous fabric swatches again were easier to disinfect compared to the nonabsorbent surfaces. However, in this case, UVC, as well as ozone treatments, failed to inactivate this fungus. Applying the smear contamination process (increased film area) on the nonabsorbent surfaces caused a significant increase in the inactivation potential of UVC; however, ozone treatment was not considerably improved. Beyond the impacts of improved surface area, the inactivation of this fungus by gaseous ozone also appears to be severely limited by mass transfer constraints (ozone gas to liquid film contaminant) on the surface. The porosity provided by the textile materials eliminates this mass transfer barrier, enabling better contact and effective inactivation. It is also worth mentioning that liquid films of the bacterial suspension were also applied to the nonabsorbent surfaces, and the same result was also observed with the droplets (complete inactivation at the utilized treatment condition). This droplet/liquid film observation is deserving of further investigation, particularly with *A. fumigatus*. Nonetheless, this finding further demonstrates the need for combined UVC and ozone treatments, particularly where hard-to-inactivate fungi like *A. fumigatus* are expected and where nonabsorbent surfaces are to be disinfected.

### 3.4 Impact of Air Circulation on UVC Disinfection

It is expected that the application of the UVC OF lamps generates some reactive oxygen species such as peroxyl radicals (ROO•) within the chamber, as shown in Figure 7. It was of interest to determine if the interaction of these radicals with the air agitation via the axial fan in the chamber had any effect on the disinfection efficiency. Table 3 (or Figure S6, Supporting Information) illustrates that the effect on the fungus is insignificant; however, the application of air circulation tends to favor the UVC disinfection process of the bacteria. This observation can be attributed to the efficient distribution of these radical species relative to the main region of their initial production. These radicals induce chemical changes to cellular structure of the microorganisms and their subsequent deactivation. Additionally, this observation may also be attributed to the drying effect that continuous air circulation produces on the bacterial cells.

**Table 3:** Effect of Air Circulation on the Inactivation Efficiency of UVC Treatment (15 cm or 0.90 mW/cm^2^ and 15 min)^a^

organism (treatment condition)	fan on	fan off
EC (PR & LR)	95.375% ± 4.07	89.675% ± 1.17
	1.44 ± 0.45	0.99 ± 0.05
SA (PR & LR)	99.717% ± 0.15	98.275% ± 0.53
	2.59 ± 0.24	1.78 ± 0.13
CA (PR)	100% ± 0	100% ± 0
AF (LR)	70.830% ± 35.47	74.290% ± 36.36

a Standard deviations are obtained from three separate runs. EC, *E. coli*; SA, *S. aureus*; CA, *C. albicans*; AF, *A. fumigatus*; PR, percentage reduction; LR, log reduction. Equivalent plots of the data in this table are presented in the Supporting Information (Figure S6).

### 3.5 Analysis of SEM Images

**Figure 9:**
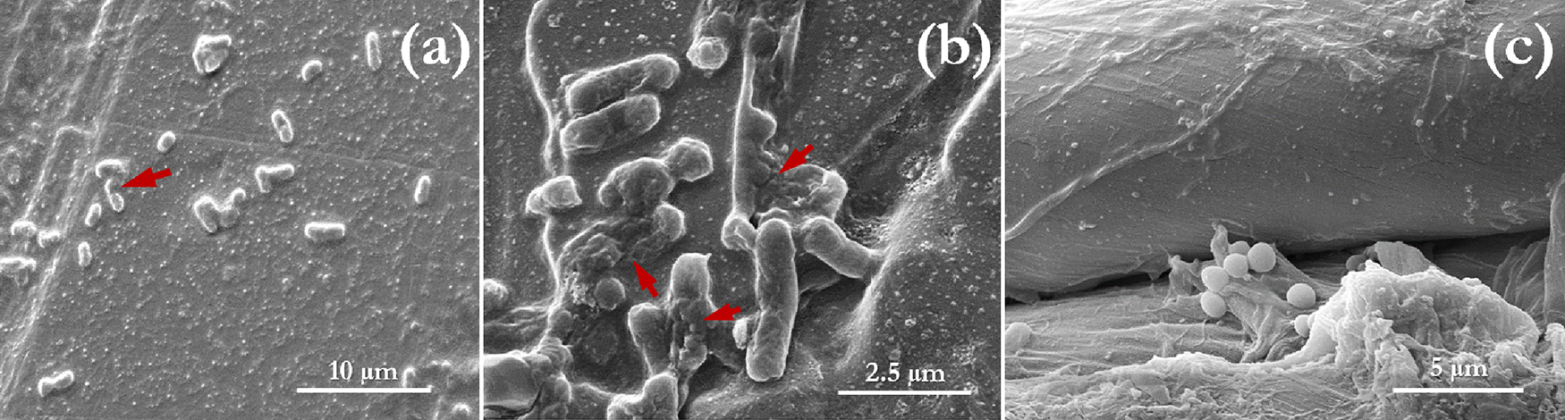
Scanning electron microscopy of (a, b) *E. coli* on the PMMA substrate; (c) *S. aureus* on the cotton-polyester fabric substrate. Red arrows indicate regions of cell damage (bacteria membrane disruption) by gaseous ozone (10 ppm for 10 min). Additional SEM images detailing the impact of gaseous ozone on different organisms can be found in other publications of the authors.^2,51^

Figure 9 illustrates the impact of ozone treatment on *E. coli* and *S. aureus* using two key materials, PMMA and the cotton-polyester fabric swatch. Although not all bacterial cells show deformation by ozone, the erosional impact of the treatment on the cell membrane can be readily observed with *E. coli*, compared to *S. aureus* (Figure 9). This can be attributed to the thicker peptidoglycan layers of the Gram-positive bacteria (*S. aureus*).

However, this does not necessarily imply that the inactivation of *S. aureus* is more challenging for ozone; rather, it is indicative of the fact that ozone’s diffusion into the cell for inactivation may occur without visible deformation, particularly because complete inactivation of *S. aureus* was observed at the applied conditions of 10 ppm and 10 min. Thus, cell lysis is not a compulsory step during the inactivation of *S. aureus* cells by ozone. A similar observation was also made in the work of Mahfoudh et al.,^62^ where they studied the inactivation of several sporulated and vegetative bacteria by dry and humidified gaseous ozone.

Despite the observation of spore inactivation in their work, the sporulated bacteria underwent no apparent structural damage (based on their SEM micrographs), eventually leading to the conclusion that diffusion and oxidation were the main mechanisms involved. This explanation also applies to *S. aureus*, as demonstrated by our SEM images. Furthermore, the SEM images showing the textile fibers reveal no structural damage (Figure 9c) at the utilized treatment conditions, an indication that the adopted concentration is safe for large-scale applications. Concentrations in the range of hundreds to thousands of ppm may not be necessary for the tested organisms, particularly in light of the potential degradative effects on the utilized material. Again, the combined application of ozone and UV, as demonstrated herein, holds great potential for the implementation of milder treatment conditions without compromising the disinfection efficiency. Nonetheless, further investigations into the critical ozone concentrations or doses for the initiation of material degradation are required, particularly during the decontamination of PPE and other materials.

### 3.6 Considerations for Large-Scale Implementation

**Figure 10:**
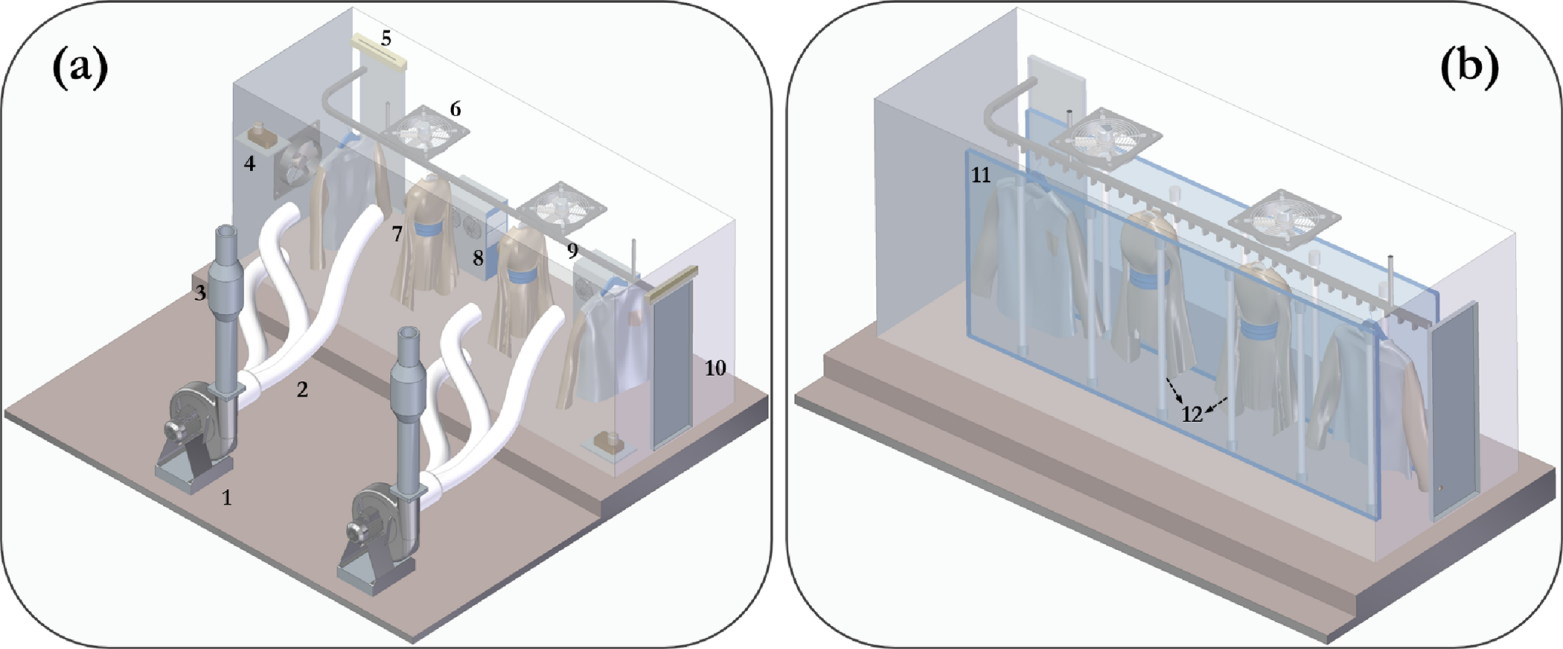
A representation of the large-scale deployment of (a) O_3_ and (b) UVC for the decontamination of clothing items. The components of the system include: (1) centrifugal fan for O_3_ removal, (2) ducting, (3) catalyst bed for O_3_ decomposition, (4) remote ozone sensor, (5) air curtain, (6) axial fans for O_3_ circulation, (7) clothing, (8) O_3_ generator, (9) conveyor, (10) O_3_ chamber, (11) UV tunnel, and (12) O_3_ free UVC lamps.

The simplicity of UV-type installations and the rapid reactivity and environmentally friendly properties of ozone make their combination very effective and thus one to explore at a large scale. Although, material degradation (incompatibility of treated substrates or chamber components) is a big challenge for ozone; the combined application of UV and ozone helps to mitigate these issues, compared to the independent application of high concentrations for the same level of microbial inactivation. Furthermore, the economics of both methods largely depend on the scale of application, and a detailed economic analysis is required to make solid conclusions. Based on the authors’ experience in clothing decontamination, higher capital costs may be required for 10 ppm ozone systems utilizing a large chamber; however, greater operating costs are likely to be incurred with UVC decontamination. A typical gaseous ozone system will involve ozone generators (most likely based on corona discharge rather than UV-based due to higher O_3_ yields), ozone sensors, oxygen concentrators, catalytic ozone destruct units, humidification and ozone circulation systems, and extraction systems for ozone removal (Figure 10). Whereas, the lamps are the critical components of UVC disinfection systems; these lamps tend to have a guaranteed high-efficiency life span of a year or two and require replacement over long operating timescales. Further considerations are highlighted in Table 4.

**Table 4:** Key Attributes of Ozone and UVC Disinfection

disinfection method	advantages	disadvantages	references
ozone	flexible application (low concentrations for longer durations, usually gives the same effect as high concentrations for shorter times)	relatively lower compatibility with some polymers, whereas the compatibility of some is still unknown	(2,49,63,64,65), this study
	environmentally friendly (decomposes to O_2_)	may produce bromates (a carcinogenic disinfection by-product) if bromine is present (e.g., during water or wastewater treatment)	
	excellent for disinfecting heat-sensitive materials	large-scale generation costs can be high since it is difficult to store	
	can be readily applied as a gas, in aqueous form and via suspended mists	requires aeration to remove excess ozone	
	excellent penetration into hard-to-reach areas of an object	inhalation causes shortness of breath, heaviness of the chest, dry throat, cough and headaches	
	effective against a wide range of organisms (sporicidal & virucidal); no activation required		
UVC	particularly effective against different fungi	poor penetration, particularly when materials of complex geometries or with considerable thickness and porosity are to be disinfected	(21,50,66), this study
	applicable in dry and wet conditions	effectiveness decreases as the distance from the UVC source increases	
	effective against a wide range of organisms; sporicidal and virucidal	high installation costs	
		induces color change and embrittlement of some plastics	
		causes severe eye and skin irritation during unprotected exposure	

## 4 Conclusions

In this study, an evaluation of the performance of UVC and ozone sterilization has been performed using four microorganisms (*E. coli*, *S. aureus*, *C. albicans*, and *A. fumigatus*) on porous and nonporous material substrates (stainless steel, polymethyl methacrylate, copper, surgical facemask, denim, and a cotton-polyester fabric).

The enhanced performance of 10 ppm of gaseous ozone over UV (0.26–15.56 mW/cm^2^) mainly pertained to the enhanced penetration over multiple stacked materials, yielding up 4 times the inactivation efficiency of UV on cotton-polyester substrates contaminated with *S. aureus*. The combined application of UV and ozone yielded an improved inactivation of all organisms except *A. fumigatus*, which demonstrated a marked sensitivity to UV treatment. At a distance of 30 cm from the lamp (0.26 mW/cm^2^), the fungus was totally inactivated after exposure for 15 min; further studies are required to thoroughly examine (at a cellular level) the marked sensitivity of *A. fumigatus* and also *C. albicans* to UVC disinfection. Of all textile materials tested (cotton-polyester, denim, and a surgical mask), denim proved the most difficult for bacterial inactivation by ozone and UV. This was attributed to the thick and multilayered fibers, which reduce the porosity and consequent penetration of ozone and UV. Thus, the decontamination of porous substrates is dependent on its structure and woven fiber architecture (in the case of textile materials).

The inactivation of the fungus on nonabsorbent surfaces was affected by the mechanism of contamination (via droplets or a smeared film of the fungal suspension). Smearing the contaminant increased the surface area for ozone action on *C. albicans*, resulting in better disinfection, compared to the droplet scenario, whereas UVC was efficient for inactivating both droplet and fully smeared forms of this fungus. Conversely, *A. fumigatus* was hard to inactivate by ozone and UV, when droplets were applied; only UVC demonstrated a considerable improvement in the disinfection efficacy when this fungus was smeared onto the nonabsorbent surfaces. This indicates that the transport mechanism of the disinfectant to the cells of the organism is not the main factor affecting the attainable disinfection efficiency; the nature and properties of the organisms themselves are key influencing factors. We also demonstrate that the diffusion of ozone into the cells of Gram-positive bacteria, and the eventual oxidation is the predominant mechanism for ozone’s inactivation of Gram-positive *S. aureus*, whereas cell lysis is more likely with Gram-negative *E. coli*. However, further work is required to ascertain the specific organelles in bacterial and fungi cells, which are prone to the oxidative effects of ozone. Furthermore, the presence of air circulation was found to favor bacterial inactivation by UVC.

This study has demonstrated the complementary attributes of ozone and UVC disinfection and the need to implement them, particularly in real industrial applications, where a myriad of microorganisms may be present. Gaseous ozone generation can be achieved via UV lamps or electric discharge and may require a more significant capital investment compared to UVC decontamination. Conversely, UVC systems are relatively straightforward to integrate into existing systems for improved decontamination efficiency but may require greater annual operating costs due to lamp replacements. A more thorough economic analysis is required to comparatively ascertain the capital and operating expenditures, using a specific case study (preferably for large-scale applications). The sterilization of medical devices is an area for which the combination of ozone and UVC constitutes a viable alternative to carcinogenic ethylene oxide currently relied on. More developments are required to enable the phase-out of this disinfectant, a global innovation challenge launched by the US Food and Drug Administration.

## Supporting Information Available

Key highlights of this study, performance of the UVC lamps utilized, enumeration methods for the bacterial and fungal contamination levels on the dipslides, and post-processed results of the data contained in Tables 1–3 of this manuscript (PDF)
