# Supplementary material for: Application of Ultraviolet-C Radiation and Gaseous Ozone for Microbial Inactivation on Different Materials
Source: ACS Omega. 2022 Nov 15;7(47):43006–21. doi: 10.1021/acsomega.2c05264 (PMC9713795; doi:10.1021/acsomega.2c05264)
Supplement: Supplementary file 1 — ao2c05264_si_001.pdf [file ao2c05264_si_001.pdf]

Application of Ultraviolet-C Radiation and Gaseous Ozone for Microbial Inactivation on Different Materials

[a,c]Emmanuel I. Epelle, [c]Andrew Macfarlane, [c]Michael Cusack, [c]Anthony Burns, [b]William G. Mackay, [a]Mostafa E. Rateb, [a]Mohammed Yaseen*

[a]*School of Computing, Engineering & Physical Sciences, University of the West of Scotland, Paisley PA1 2BE, United Kingdom*

[b]*School of Health & Life Sciences, University of the West of Scotland, Paisley PA1 2BE, United Kingdom*

[c]*ACS Clothing, 6 Dovecote Road Central Point Logistics Park ML1 4GP, United Kingdom*

Supporting Information

Study highlights

- UVC radiation and 10 ppm O_3 are analysed for their decontamination efficacies on stainless steel, PMMA, copper, surgical facemask, denim, and a cotton-polyester fabric.
- The combined application of UV and O_3 improved inactivation efficiency.
- *A. fumigatus* and *C. albicans* demonstrated a marked sensitivity to UV treatment.
- Ozone possesses better penetration efficiency compared to UVC radiation in a stack of porous fabric swatches.
- Spread-out films of the fungi are easier to inactivate than droplets on the material surfaces.
- UVC and O_3 decontaminated the substrates, without affecting their structural integrity.

S1. Methodology

S1.1. Performance of UVC lamps

Fig. S1 illustrates the performance of the UVC lamps used in this study. According to Fig. S1b, the 185 and 254 nm wavelengths are the main spectral lines emitted, and the lamps reach optimal performance, within 2 mins after they are switched on (Fig. S1c). The intensity-distance profile shows a rapid decrease between 0 and 5 cm (Fig. S1d); as shown in the main manuscript, we correlate this intensity profile to the disinfection efficiency attained for the different organisms. As observed in Fig. S1b, other wavelengths (280 nm) are emitted by the lamp; but possess very minimal disinfection capabilities, as demonstrated in the work of Schuit et al.[1]. At the UVC lamp's surface, the intensity of the 254 nm spectral line is 30 mW/cm^2 (Ref.[2]); whereas, the intensities at 1, 5, 15 and 30 cm from the lamp, as determined by the UVC detector are shown in Fig. S1d and Table S1. In Fig S1a, the typical profile of an ozone disinfection cycle involving the generation phase, the stabilisation phase at the desired concentration and the decomposition phase via rapid extraction from the chamber are depicted. Curve 1 in Fig. S1a, demonstrates the ozone-generating capacity of a single ozone-producing lamp. At approximately 4 mins, when the lamp is turned off, ozone's auto-decomposition commences, and the concentration is observed to slowly decrease over a 3-min duration. For the accurate stabilisation of the ozone concentration in the chamber, an optimised on/off sequence was established before commencing the experiments.

The profile of 1 OG and 1 OF lamp, simultaneously switched on is depicted as Curve 2 (Fig. S1a). It can be observed that the OF lamp, limits the ozone production potential of the OG lamp by reducing the ozone generation rate and eventually the peak concentration at the 4-min mark. After the generation phase, the OG lamp is turned off, whereas, the OF lamp is left on. The result of this is a more rapid ozone decomposition rate relative to Curve 1. This demonstrates the ozone-destroying capability of the OF lamps, which principally generate 254 nm photons. After this phase, the extraction phase is initiated using a centrifugal fan. Curve 3 (Fig. S1a) shows the increased effect of ozone decomposition, using 1 OG lamp and 2 OF lamps; thus demonstrating the effectiveness of OF lamps for ozone decomposition, while simultaneously providing disinfection capabilities. Curve 4 shows the ozone generating capability of 3 OG lamps operating simultaneously, attaining up to 15 ppm in 2 mins of operation. The inclusion of Curve 4 in Fig. S1a is only to demonstrate that ozone concentrations >> 10 ppm (the nominal concentration utilised herein) can be attained in our apparatus. With 4 OG lamps, 10 ppm ozone concentration can be achieved in less than a minute.

Table S1: UV intensity and doses at different distances from the UV lamps.

Distance from the light source (cm)	Intensity (mW/cm²)	UVC dose (mJ/cm²) for 5-min exposure	UVC dose (mJ/cm²) for 10-min exposure	UVC dose (mJ/cm²) for 15-min exposure
1	15.56	4668	9336	14004
5	3.22	966	1932	2898
15	0.90	270	540	810
30	0.26	78	156	234
60	0.077	23.1	46.2	69.3

The UVC intensities are measured using robust UVC detectors with very high repeatability and reproducibility (relative standard deviation of < 2%). UVC dose = UVC intensity × Exposure time

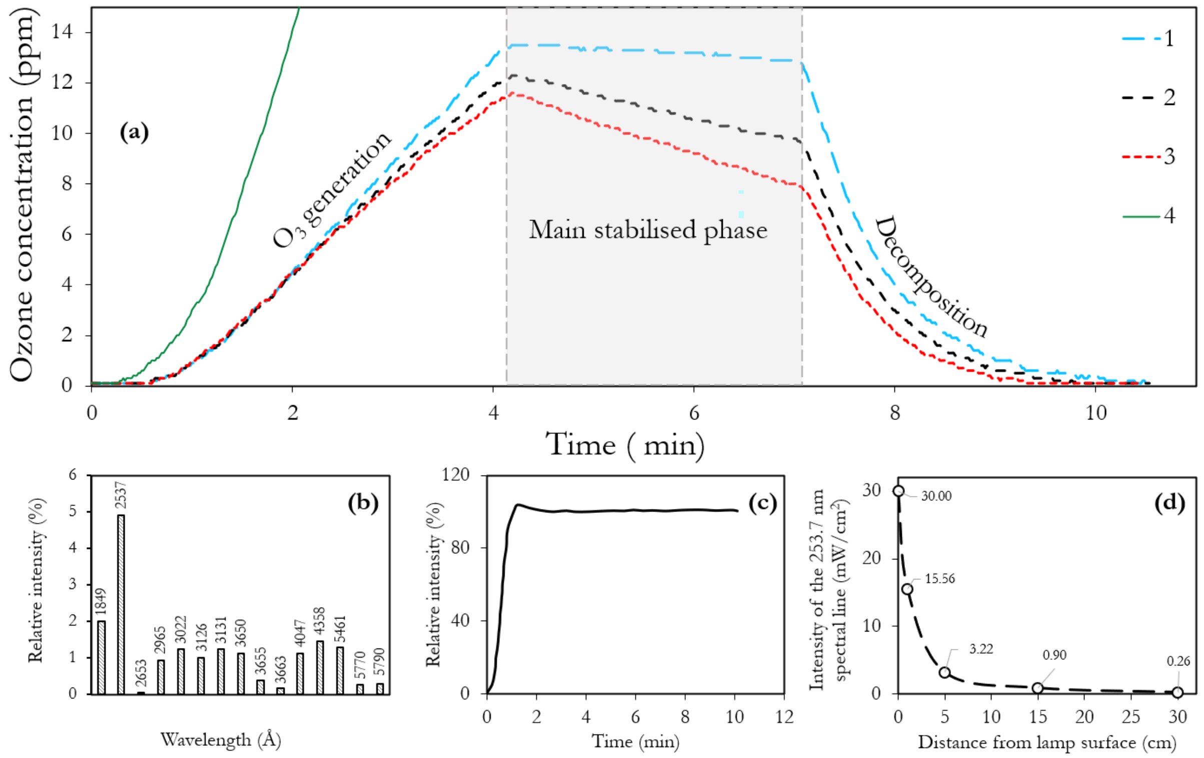

Figure S1: (a) Ozone generation cycles showing the impact of OF lamps on the generation, stabilisation and assisted-decomposition phases. 1 OG lamp – Curve 1; 1 OG lamp and 1 OF lamp – Curve 2; 1 OG lamp and 2 OF lamps – Curve 3; 3 OG lamps – Curve 4. (b) Wavelength spectrogram of the OG lamps; the wavelength spectrogram of the OF lamps is similar but without the 184.9 nm spectral line [2]. (c) Stabilisation time from lamp start-up. (d) UVC intensities at the OF lamp's surface and at 1, 5, 15 and 30 cm from the lamp, showing exponential decrease; the intensity of the 184.9 nm spectral line of the OG lamps was 1.3 mW/cm^2.

S1.2. Evaluating the number of bacterial colonies and fungal area fraction

The following set of MATLAB codes (Fig. S2) were utilised to compute the contamination levels on the dipslide for the various organisms worked with, and to post-process the results.

```matlab
% Contour plots for bacterial and fungal inactivation
clc; clear
% Log reduction (LR) data
DataLR    = xlsread('SAResults-AF_NoCol.xlsx','(UV+O3)NC','AB6:AE17');
% Percentage reduction (PR) data
DataPR    = xlsread('SAResults-AF_NoCol.xlsx','(UV+O3)NC','AB20:AE31');
Intensity =  DataPR(:,1);
Distance  =  DataPR(:,2);
Time      =  DataPR(:,3);
LRorPR    =  DataPR(:,4);
x = Distance; y = Time; z = LRorPR;

dt = delaunayTriangulation(x,y);
tri = dt.ConnectivityList;
xi = dt.Points(:,1);
yi = dt.Points(:,2);
F = scatteredInterpolant(x,y,z);
zi = F(xi,yi) ;
P = trisurf(tri,xi,yi,zi);
caz = 12.9481; cel = 10.6612;
view(caz,cel);
shading interp;
grid on;
ztickformat('%.1f');
h = colorbar;
colormap jet;
t=get(h,'Limits');
ta = [0, 4];
T=linspace(t(1),t(2),5);
set(h,'Ticks',T);
TL=arrayfun(@(x) sprintf('%.0f',x),T,'un',0);
set(h,'TickLabels',TL);
set(gcf,'color','w');
```

1

```matlab
%% Number of colonies on an agar slide (Evaluation of bacterial
contamination)
clc
% BW22 is the generated matrix of the image (contaminated dipslide)
% after it is read into Matlab
% Always ensure that BW22 on the RHS is updated
[BW_outCol,propertiesCol] = filterRegions(BW22);
MCol = [propertiesCol.Area]';
SCol = size(MCol);

%% CFUs/cm2 of agar slide (Evaluation of bacterial contamination)
No_of_Col = SCol(1)
CFUs_per_cmsq = (No_of_Col * 2)/10
```

2

```matlab
%% Cummulative area of contaminated region (Fungal contamination level)
clc
% BW21: Contaminated dipslide image read into Matlab
% BW22: Sterile dipslide image read into Matlab

% Always ensure that BW? on the RHS is updated
[BW_out,properties] = filterRegions(BW22);
M = [properties.Area]';
Sum_of_cont_Area = sum(M)

%% Cummulative area of entire slide
% Always ensure that BW? on the RHS is updated
[BW_out1,properties1] = filterRegions(BW21);
M1 = [properties1.Area]';
Area_of_Slide = sum(M1)

%% Area fraction of fangal growth
Area_Fraction = (Sum_of_cont_Area/Area_of_Slide)*100
```

3

```matlab
function [BW_out,properties] = filterRegions(BW_in)
%  filterRegions  Filter BW image generated from imageRegionAnalyzer app.
%  [BW_OUT,PROPERTIES] = filterRegions(BW_IN) filters binary image BW_IN
%  using auto-generated code from the imageRegionAnalyzer app. BW_OUT has
%  had all of the options and filtering selections that were specified in
%  imageRegionAnalyzer applied to it. The PROPERTIES structure contains the
%  attributes of BW_out that were visible in the app.
%-----------------------------------------------------------

BW_out = BW_in;

% Get properties.
properties = regionprops(BW_out, {'Area', 'Eccentricity', 'EquivDiameter', 'EulerNumber', 'MajorAxisLength', 'MinorAxisLength', 'Orientation',
'Perimeter'});

% Sort the properties.
properties = sortProperties(properties, 'Area');
% Uncomment the following line to return the properties in a table.
% properties = struct2table(properties);

function properties = sortProperties(properties, sortField)

% Compute the sort order of the structure based on the sort field.
[~,idx] = sort([properties.(sortField)], 'descend');

% Reorder the entire structure.
properties = properties(idx);
```

2 & 3

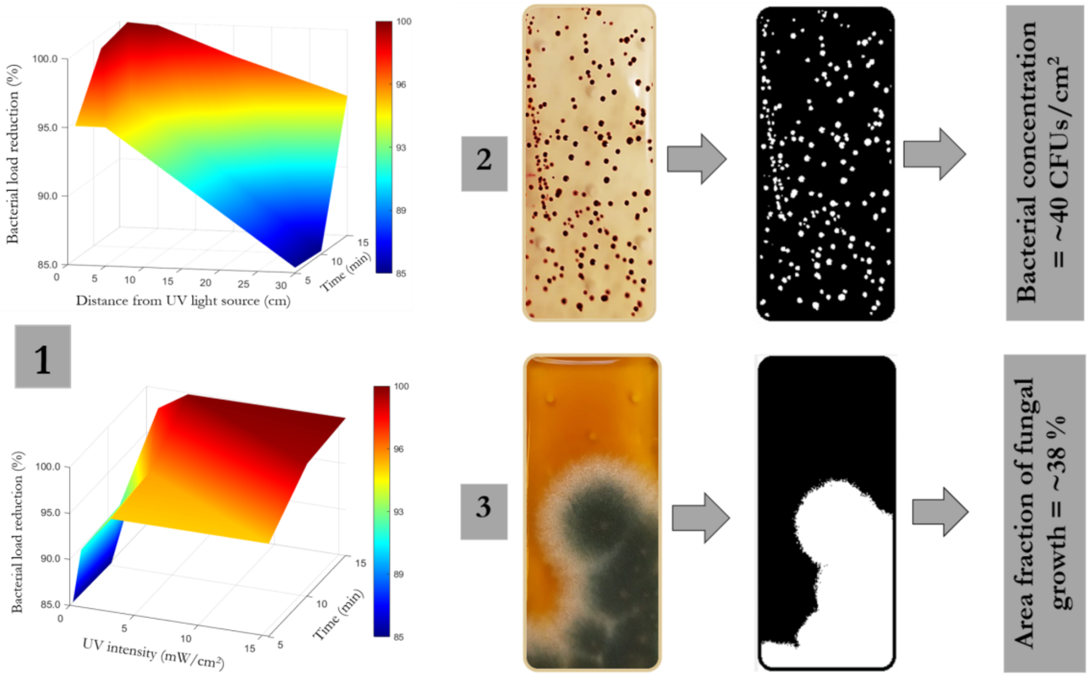

Figure S2: Evaluating the microbial contamination efficiency.

S2. Post-processed results

Figs. S3-S6 are graphical illustrations of the data contained in Tables 1-3 of the main manuscript. This is aimed at helping the reader to better interpret the data.

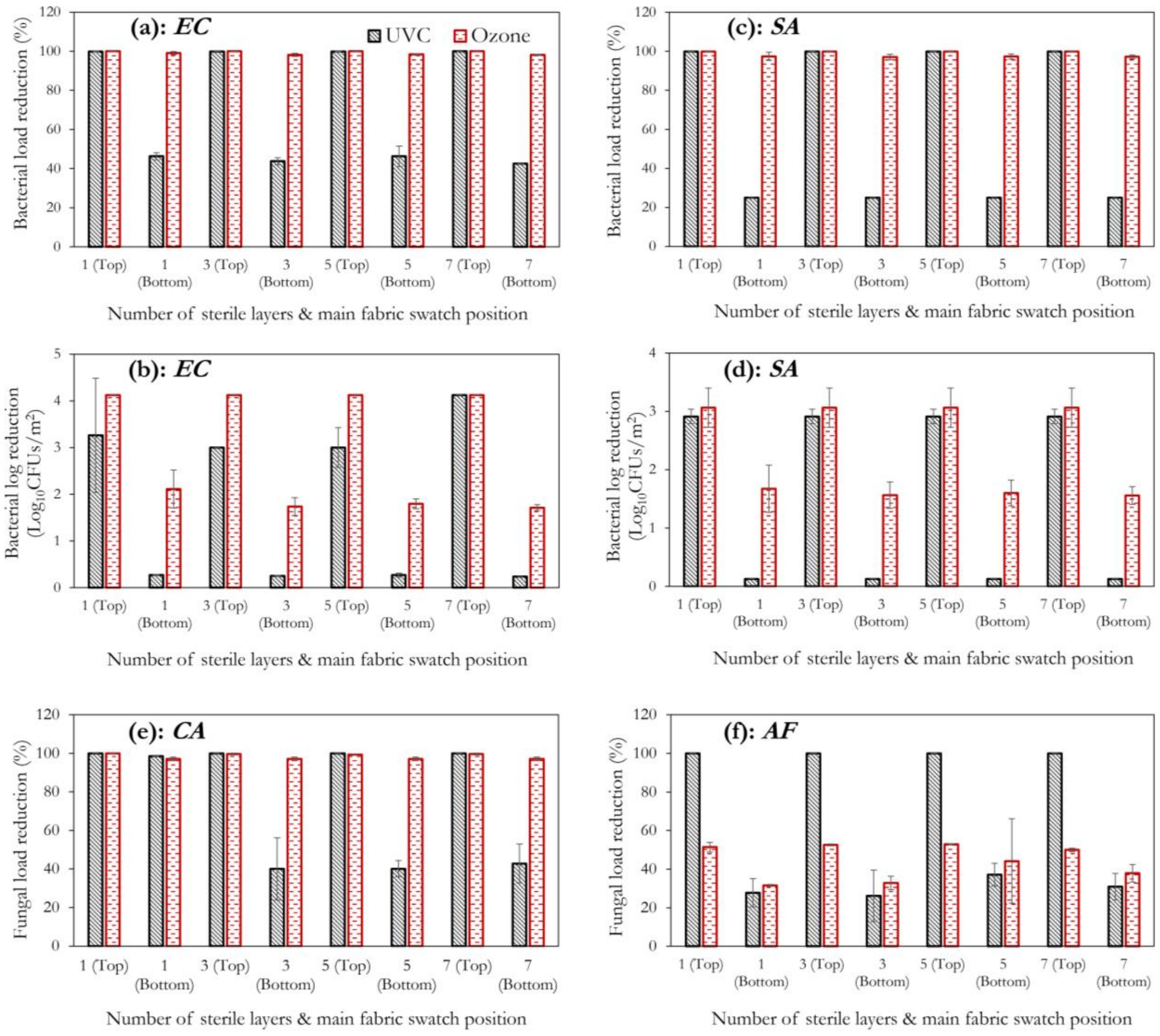

Figure S3: Effect of the number of sterile layers on the penetration of UVC (1 cm or 15.56 mW/cm^2 and 15 mins) and ozone (10 ppm, 15 mins) for microbial inactivation of (a, b) *E. coli* (*EC*); (c, d) *S. aureus* (*SA*); (e) *C. albicans* (*CA*); (f) *A. fumigatus* (*AF*). Error bars represent the standard deviations of 3 separate runs.

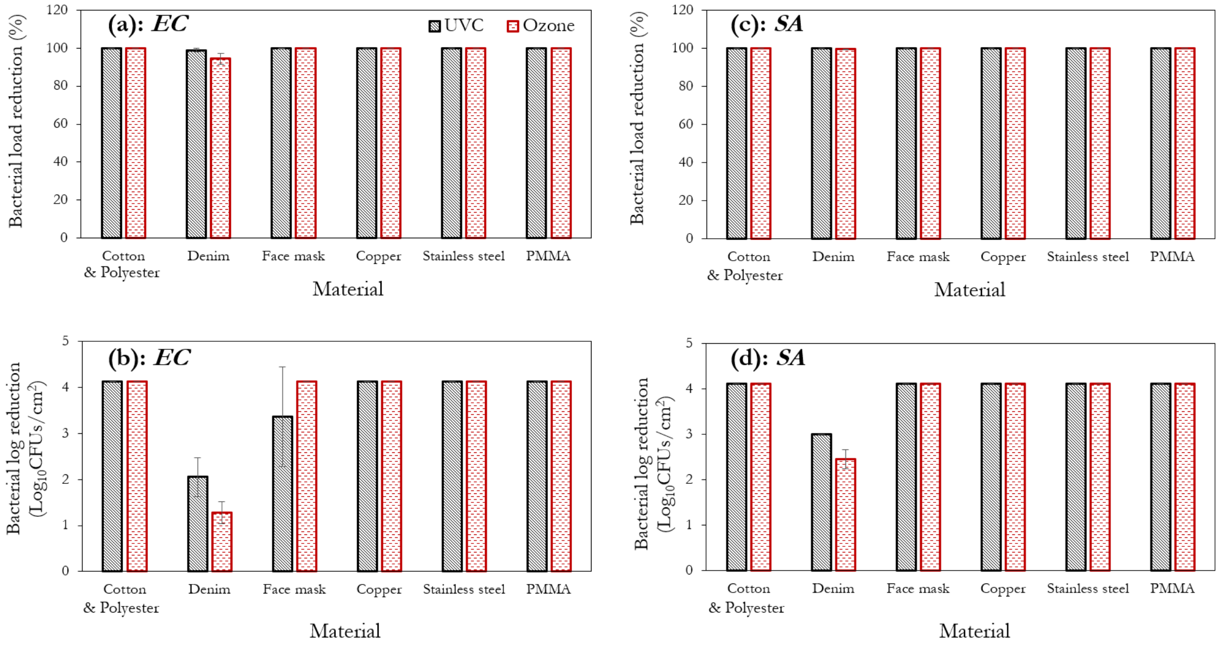

Figure S4: Effect of material type on the disinfection efficacy of ozone (10 ppm 15 mins) and UVC (1 cm or 15.56 mW/cm^2 and 15 mins) treatments for the different bacteria (a, b) *E. coli* (*EC*); (c, d) *S. aureus* (*SA*). Error bars represent the standard deviations of 3 separate runs.

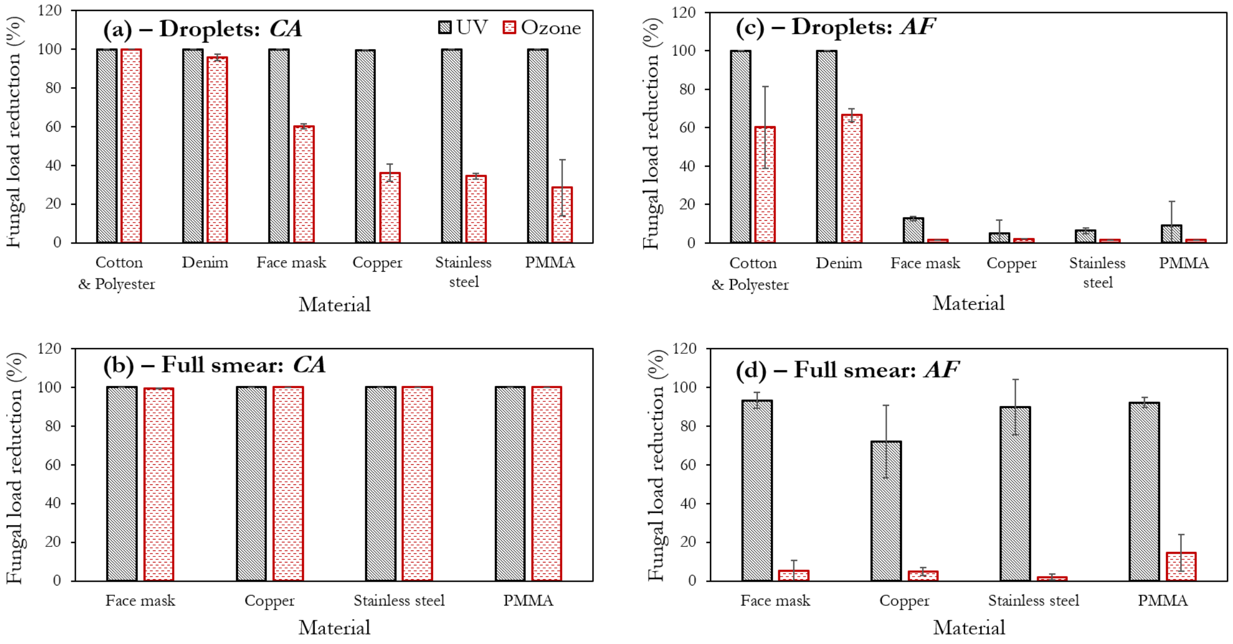

Figure S5: Effect of material type on the disinfection efficacy of ozone (10 ppm 15 mins) and UVC (1 cm or 15.56 mW/cm^2 and 15 mins) treatments for the different fungi (a, b) *C. albicans* (*CA*) and (c, d) *A. fumigatus* (*AF*). Error bars represent the standard deviations of 3 separate runs.

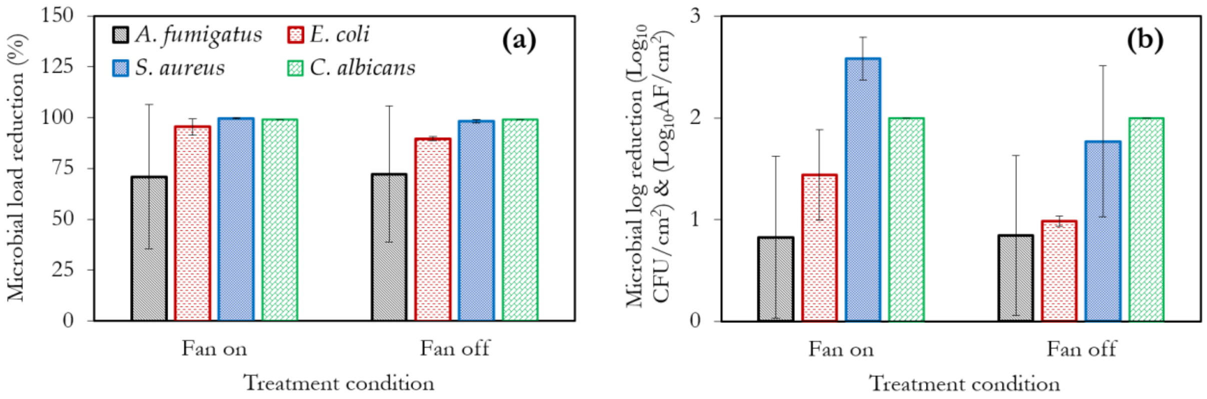

Figure S6: Effect of air circulation on the inactivation efficiency of UVC treatment (15 cm or 0.90 mW/cm^2 and 15 mins); (a) shows the percentage reduction, whereas (b) shows the log reduction. Error bars represent the standard deviations of 3 separate runs.

S3. References

(1) Schuit, M. A.; Larason, T. C.; Krause, M. L.; Green, B. M.; Holland, B. P.; Wood, S. P.; Grantham, S.; Zong, Y.; Zarobila, C. J.; Freeburger, D. L. SARS-CoV-2 Inactivation by Ultraviolet Radiation and Visible Light Is Dependent on Wavelength and Sample Matrix. *J. Photochem. Photobiol. B Biol.* **2022**, *233*, 112503.

(2) Jelight Company Inc. *Low Pressure Mercury Vapor Ozone Producing Double-Bore Lamp*. https://www.jelight.com/ (accessed 2022-03-25).
